# Targeting osteoclasts for treatment of high-risk B-cell acute lymphoblastic leukemia

**DOI:** 10.1038/s41408-025-01239-3

**Published:** 2025-02-27

**Authors:** Rishi S. Kotecha, Sarah M. Trinder, Anastasia M. Hughes, Benjamin H. Mullin, Sarah Rashid, Jinbo Yuan, Jiake Xu, Owen Duncan, Patrycja Skut, Grace-Alyssa Chua, Sajla Singh, Joyce Oommen, Richard B. Lock, Ursula R. Kees, Sebastien Malinge, Vincent Kuek, Laurence C. Cheung

**Affiliations:** 1 Leukaemia Translational Research Laboratory, WA Kids Cancer Centre, The Kids Research Institute Australia, Perth, WA Australia; 2 Curtin Medical School, Curtin University, Perth, WA Australia; 3 Department of Clinical Haematology, Oncology, Blood and Marrow Transplantation, Perth Children’s Hospital, Perth, WA Australia; 4 Medical School, University of Western Australia, Perth, WA Australia; 5 School of Biomedical Sciences, University of Western Australia, Perth, WA Australia; 6 Department of Endocrinology and Diabetes, Sir Charles Gairdner Hospital, Perth, WA Australia; 7 Shenzhen Institute of Advanced Technology, Chinese Academy of Sciences, Shenzhen, China; 8 Centre for Microscopy, Characterisation and Analysis, University of Western Australia, Perth, WA Australia; 9 Children’s Cancer Institute, Lowy Cancer Research Centre, University of New South Wales, Sydney, NSW Australia; 10 School of Clinical Medicine, UNSW Medicine & Health, University of New South Wales, Sydney, NSW Australia; 11 UNSW Centre for Childhood Cancer Research, University of New South Wales, Sydney, NSW Australia; 12 Curtin Medical Research Institute, Curtin University, Perth, WA Australia

**Keywords:** Acute lymphocytic leukaemia, Cancer microenvironment

B-cell acute lymphoblastic leukemia (B-ALL) is the most common childhood cancer. Outcomes have improved significantly over the last 50 years, with 5-year overall survival approaching 90%, but certain subgroups have inferior prognoses, especially those with high-risk genetic alterations in their leukemia cells, such as *BCR-ABL1* or relapsed disease [1]. The tumor microenvironment is known to play an integral role in the development and progression of cancer. For hematological malignancies, the bone marrow (BM) microenvironment serves as the site of initiation, progression, and relapse. It consists of different cell types, including hematopoietic stem and progenitor cells, immune cells, fibroblasts, endothelial cells, osteoblasts, and osteoclasts. Clinical studies in children diagnosed with ALL have demonstrated bone loss and changes in the BM microenvironment during disease development [2, 3], suggesting that restoring the healthy BM microenvironment represents an attractive therapeutic avenue, especially for children with high-risk leukemia where dose-limiting toxicities of conventional chemotherapeutic agents have prevented further improvement.

## Results

**Fig. 1:**
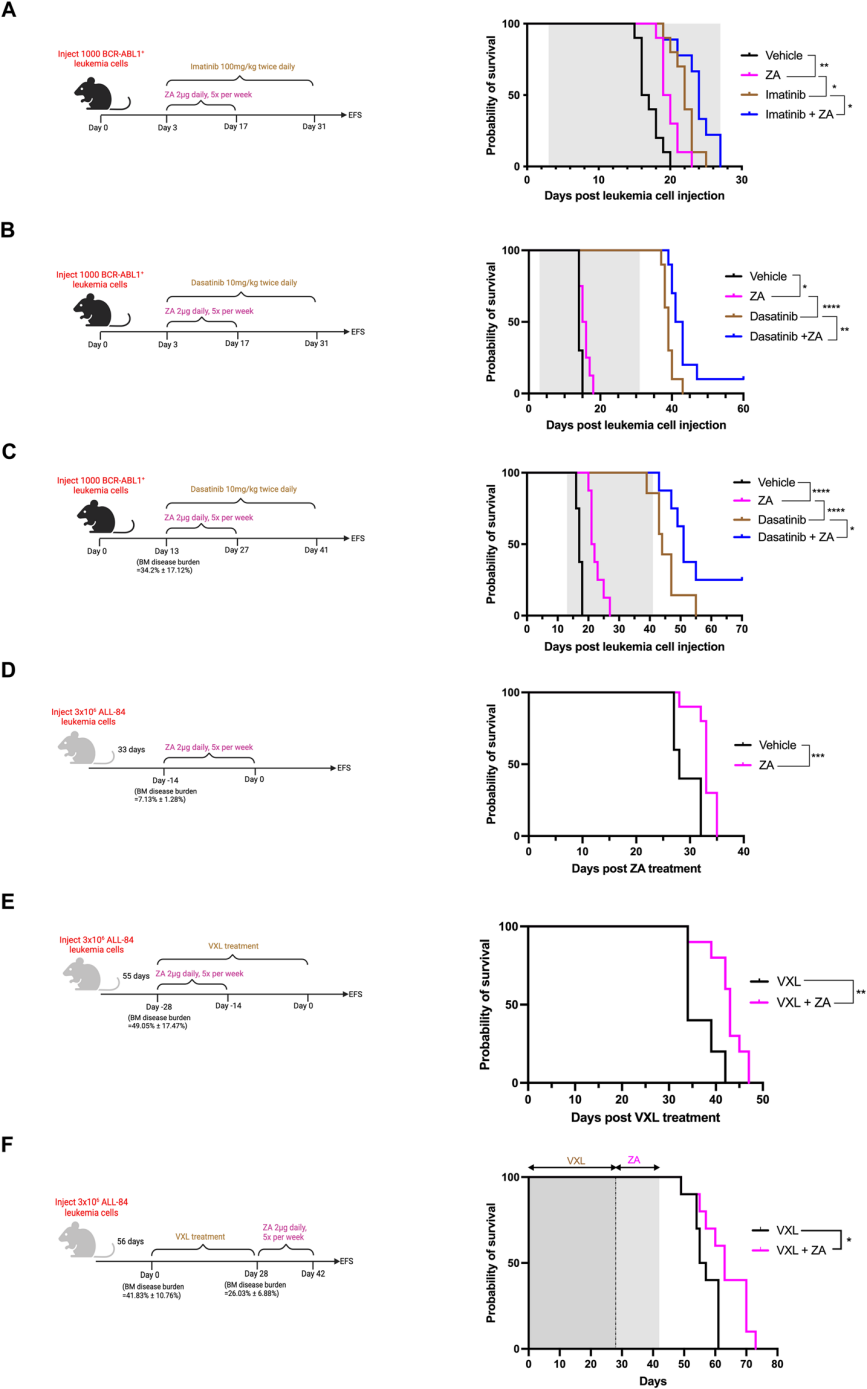
Zoledronic acid significantly prolongs survival when combined with conventional therapy or administered after induction therapy. Left, schematic diagram of the treatment schedule. Right, Kaplan–Meier curves representing event-free survival of leukemia-bearing mice. **A** Mice were treated with zoledronic acid (ZA) and imatinib commencing 3 days post-BCR-ABL1^+^ leukemia cell injection (low disease burden setting). Mice succumbed to disease before completion of the imatinib treatment (*n* = 9–10 mice per group). **B** Mice were treated with ZA and dasatinib commencing 3 days post-BCR-ABL1^+^ leukemia cell injection (low disease burden setting). One mouse survived more than 180 days post-ZA and dasatinib treatment (*n* = 8–10 mice per group). **C** Mice were treated with ZA and dasatinib commencing 13 days post-BCR-ABL1^+^ leukemia cell injection when the disease burden in the bone marrow was 34.2 ± 17.12% (high disease burden setting). Two mice survived more than 180 days post-ZA and dasatinib treatment (*n* = 8 mice per group). **D** Mice were treated with ZA for 2 weeks commencing 33 days post-ALL-84 leukemia cell injection when the disease burden in the bone marrow was 7.13 ± 1.28% (*n* = 10 mice per group). **E** Mice were treated with ZA for 2 weeks and VXL (vincristine, dexamethasone, and l-asparaginase) for 4 weeks commencing 55 days post-ALL-84 leukemia cell injection when the disease burden in the bone marrow was 49.05 ± 17.47% (*n* = 10 mice per group). **F** Mice were treated with VXL for 4 weeks commencing 56 days post-ALL-84 leukemia cell injection when the disease burden in the bone marrow was 41.83 ± 10.76% followed by ZA treatment for 2 weeks (*n* = 10 mice per group). Throughout, the gray-shaded areas indicate the treatment periods. *p < 0.05, **p < 0.01, ***p < 0.001 and ****p < 0.0001.

Using a murine BCR-ABL1^+^ B-ALL syngeneic model, we previously reported that osteoclasts are responsible for leukemia-induced bone loss [4]. In this study, we aimed to evaluate the efficacy of targeting osteoclasts using zoledronic acid (ZA) and tested the hypothesis that combination therapy using ZA and conventional therapy is more effective than conventional therapy alone (Supplementary Methods). Tyrosine kinase inhibitors comprise standard treatment for BCR-ABL1^+^ B-ALL and thus ZA was investigated in combination with imatinib or dasatinib. First, we administered the drugs 3 days following the injection of leukemia cells, at a time when the disease burden was undetectable in the BM. ZA alone was found to significantly enhance the survival of leukemia-bearing mice compared to vehicle, and further improvements were achieved in combination with imatinib or dasatinib compared to imatinib or dasatinib alone (Fig. 1A, B). Next, we examined the efficacy of ZA in a high disease burden setting (34.2 ± 17.12%). ZA alone or in combination with dasatinib significantly improved survival in the presence of high leukemia burden, compared to treatment with vehicle or dasatinib alone, respectively (Fig. 1C).

Micro-CT analysis revealed that ZA significantly reversed leukemia-induced trabecular bone loss and cortical thinning (Supplementary Fig. 1). TRAP staining of bone sections showed a significant reduction in the number of TRAP+ multinucleated osteoclasts upon completion of 2 and 4 weeks of therapy (Supplementary Fig. 2A–C) and also when mice succumbed to disease after treatment (Supplementary Fig. 2D–F). Dasatinib is known to dysregulate bone remodeling by inhibiting osteoclast activity and abrogating osteoclast formation [5, 6]. While this was also observed in our study, ZA was shown to be a more potent osteoclast inhibitor than dasatinib (Supplementary Fig. 2B, C, E, F). There were no significant changes to other BM monocyte-lineage populations with the addition of ZA to dasatinib therapy (Supplementary Fig. 3).

To further consolidate these findings, we evaluated the efficacy of ZA in the ALL-84 patient-derived xenograft model, which was derived from a 14-year-old boy with relapsed B-ALL and expresses high levels of *Rankl* [7]. Using this independent model, we again demonstrated severe bone loss during leukemia development (Supplementary Fig. 4). ZA injections started when the disease burden in the BM was 7.13 ± 1.28%. We found that ZA significantly improved survival compared to vehicle (Fig. 1D). Similar to the BCR-ABL1^+^ B-ALL syngeneic mouse model, micro-CT analysis revealed that ZA significantly reversed leukemia-induced trabecular bone loss and cortical thinning (Supplementary Fig. 5).

Next, we explored the efficacy of ZA in combination with standard chemotherapeutic agents used during induction therapy for children with B-ALL, namely vincristine, dexamethasone, and l-asparaginase (VXL). In a high disease burden setting (49.05 ± 17.47%), we showed a significant survival advantage in mice receiving ZA in combination with VXL compared to mice treated with VXL alone (Fig. 1E). Furthermore, micro-CT analysis (Supplementary Fig. 6) and TRAP staining of bone sections (Supplementary Fig. 7) revealed that ZA reversed bone loss.

We next explored the use of ZA after induction therapy. First, to evaluate whether ZA could inadvertently promote relapse, mice were treated with VXL and dasatinib commencing 3 days post-BCR-ABL1^+^ leukemia cell injection (Supplementary Fig. 8A). After 4 weeks of treatment, the leukemia-bearing mice were in complete remission with no disease detected in the BM, spleen, and blood. ZA was administered immediately after induction therapy. Micro-CT analysis again demonstrated that treatment with ZA significantly reversed trabecular bone loss and cortical thinning (Supplementary Fig. 8B, C). Importantly, treatment with ZA, either for two weeks or continuously following induction, did not promote leukemia relapse (Supplementary Fig. 8D, E).

Next, we evaluated the post-induction use of ZA on survival. Using the ALL-84 model, mice received 4 weeks of VXL induction therapy when the disease burden in the BM was 41.83 ± 10.76%. ZA was given immediately after induction therapy for 2 weeks when the disease burden in the BM was 26.03 ± 6.88%. We found that ZA significantly improved survival compared to vehicle (Fig. 1F). In addition, micro-CT analysis confirmed that post-induction use of ZA reversed bone loss (Supplementary Fig. 9).

In the clinical setting, we report three vignettes that suggest the feasibility and safety of incorporating ZA early into leukemia therapy. Three children presented with a bone-symptomatic phenotype of B-ALL, with diffuse marrow infiltration and multi-level vertebral body crush fractures seen on imaging of their spines (Supplementary Figs. 10–12). ZA was concurrently administered during induction or consolidation therapy. Detailed descriptions of the clinical cases can be found in the Supplementary Results. In all three cases, ZA was well tolerated without any significant adverse effects, suggesting the feasibility of safely administering ZA in combination with chemotherapy during the early phases of treatment in children with ALL.

**Fig. 2:**
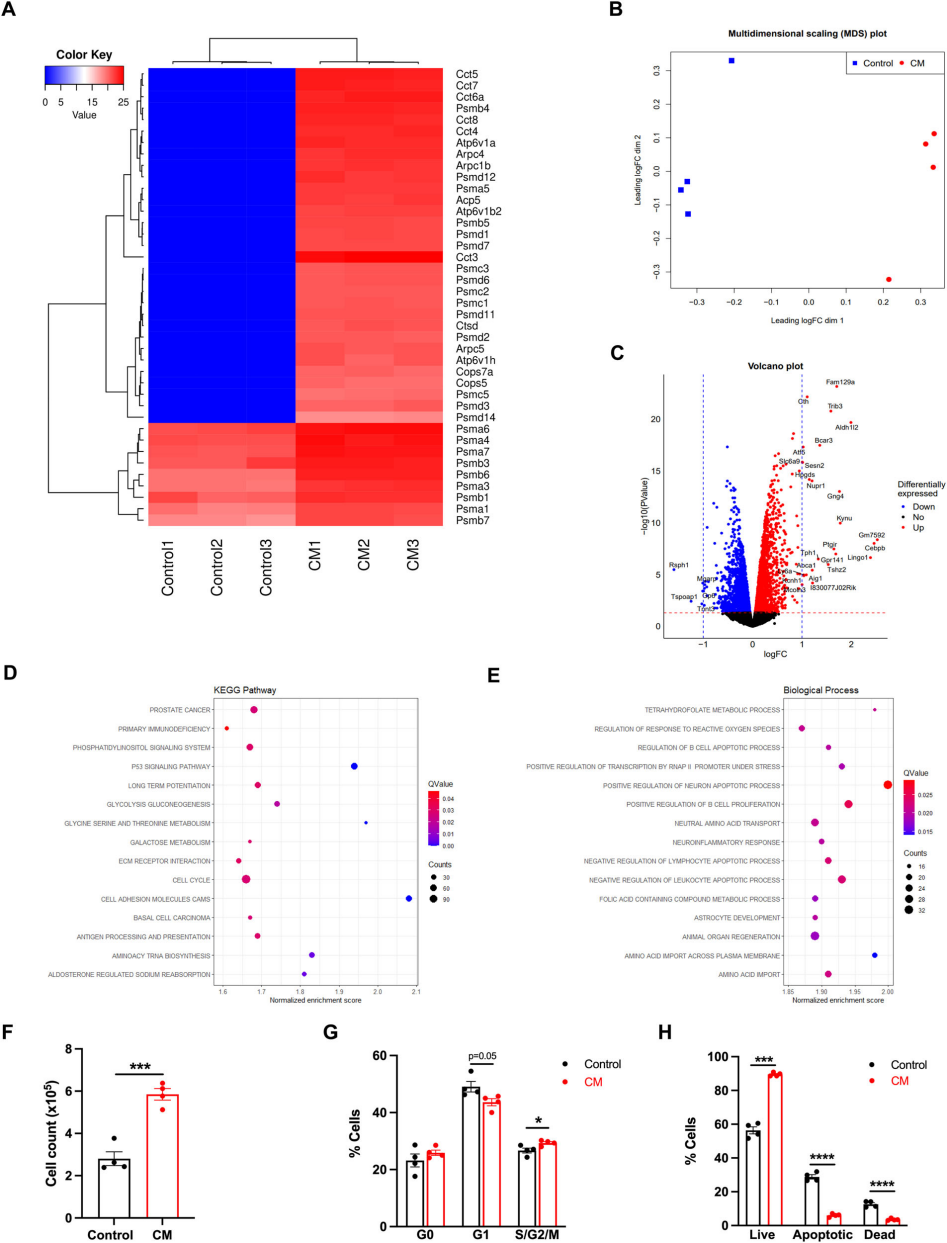
Soluble factors from osteoclasts promote leukemia cell proliferation and survival. **A** Proteomic analysis of the supernatant from differentiated RAW264.7 cells compared to controls (*n* = 3 per group). The value refers to the label-free quantitation intensity. **B** Multidimensional scaling analysis of global gene expression in leukemia cells cultured with conditioned media (CM) (*n* = 4) and control media (*n* = 4). **C** Volcano plot highlighting the top differentially expressed genes of leukemia cells following culture with CM and control media. **D**, **E** Gene set enrichment analysis comparing RNA sequencing-generated global transcriptomes of leukemia cells in CM (*n* = 4) vs. leukemia cells in control media (*n* = 4) by **D** KEGG pathway and **E** GO Biological Process annotations. Significance was assigned by *q*-value < 0.05. **F** Number of leukemia cells after culture with CM and control media for 3 days (*n* = 4 per group). **G** Cell cycle analysis of leukemia cells cultured with CM and control media for 3 days (*n* = 4 per group). **H** Apoptosis assay of leukemia cells in CM and control media for 3 days (*n* = 4 per group). **F–H** Error bars are represented as mean ± SEM. *p < 0.05, ***p < 0.001, ****p < 0.0001 or the precise *p*-value where indicated.

Finally, we investigated mechanisms of crosstalk between osteoclasts and leukemia cells. The murine RAW264.7 cell line was used as an osteoclast source. RAW264.7 cells readily differentiated into TRAP+ multinucleated osteoclasts in the presence of RANKL (Supplementary Fig. 13). Proteomic analysis by mass spectrometry confirmed that conditioned media (CM) from differentiated RAW264.7 cells was enriched in various proteins, including the proteasome, ACP5, cathepsin D, T-complex protein 1, Arp2/3 complex, V-ATPase, and coatomer protein 1, that are known to be secreted by osteoclasts (Fig. 2A) [8].

The CM was used to study the role of osteoclasts on BCR-ABL1^+^ PER-M60 leukemia cells [4]. Using RNA sequencing, we compared the molecular profile of leukemia cells cultured with CM or control media. There were 30 differentially expressed genes identified at the 5% false discovery rate with a log fold-change value ≥1 or ≤–1 (Fig. 2B, C; Supplementary Fig. 14A). Most of these genes were upregulated rather than downregulated in leukemia cells cultured in CM. The most deregulated gene, Fam129a (*p* = 6.91 × 10^−24^), is known to play a role in modulating p53-mediated apoptosis, and overexpression of Fam129a has been associated with many types of cancer [9, 10]. KEGG pathway analysis revealed significant upregulation in the p53 signaling, cell cycle, cell adhesion molecules, and extracellular matrix receptor interaction pathways in leukemia cells cultured with CM compared to control (Fig. 2D; Supplementary Fig. 14B). GO Biological Process analysis showed significant upregulation on positive regulation of B-cell proliferation as well as negative regulation of lymphocyte and leukocyte apoptotic process in leukemia cells cultured with CM compared to control (Fig. 2E; Supplementary Fig. 14C).

To examine the cellular effect of osteoclasts on leukemia cells, we showed that osteoclast-derived CM significantly increased B-ALL cell proliferation, demonstrated by significantly increased cell numbers (Fig. 2F) and increased S/G2/M phases *via* cell cycle analyses (Fig. 2G). Using annexin V and DAPI staining, we further demonstrated significantly more live cells and fewer apoptotic and dead cells when leukemia cells were cultured with CM compared to control (Fig. 2H). Together, our results reveal that soluble factors produced by osteoclasts strongly promote B-ALL cell proliferation and survival.

## Discussion

In this study, we reveal an important role for osteoclasts during B-ALL development. Our findings demonstrate a multi-component crosstalk between osteoclasts and leukemia cells, and that soluble factors produced by osteoclasts promote B-ALL cell proliferation and survival. In addition, our results are in line with the reported role of osteoclasts in B-cell development and formation of hematopoietic stem cell niches in the BM [11, 12]. Osteoclasts are involved in bone metastasis of solid tumors [13], and a transient increase in osteoclast numbers during the development of acute myeloid leukemia has been reported [14], highlighting osteoclasts as an attractive therapeutic target for many different cancer types.

Skeletal abnormalities are well recognized in children with ALL at diagnosis, with fractures, osteopenia, and periosteal reactions frequently observed [3]. Unanswered questions remain as to whether skeletal abnormalities at diagnosis should be treated, whether bisphosphonates have any impact on leukemia progression, and if they are clinically safe to use in conjunction with chemotherapy during the early phases of therapy. Our findings indicate that ZA has the ability to provide dual clinical benefit in terms of restoring bone health and improving survival in high-risk B-ALL. There is also potential for ZA to derive further clinical benefit, with a preclinical study reporting that ZA may prevent osteonecrosis, a well-recognized complication of childhood ALL treatment, if it is initiated early in therapy [15]. Taken together, our results suggest that targeting osteoclasts is a promising therapeutic strategy for children with high-risk B-ALL, and ZA in combination with conventional therapy warrants further evaluation in future clinical trials.

## Supplementary information

Supplementary Figures 1 to 14

Supplementary Results

Supplementary Methods

## Data Availability

Raw sequencing data is available via the Gene Expression Omnibus (GEO) database under the accession number GSE220262.
