# Supplementary material for: Targeting osteoclasts for treatment of high-risk B-cell acute lymphoblastic leukemia
Source: Blood Cancer J. 2025 Feb 27;15(1):25. doi: 10.1038/s41408-025-01239-3 (PMC11868389; doi:10.1038/s41408-025-01239-3)
Supplement: Supplementary file 1 — Supplementary Figures 1 to 14 [file 41408_2025_1239_MOESM1_ESM.pdf]

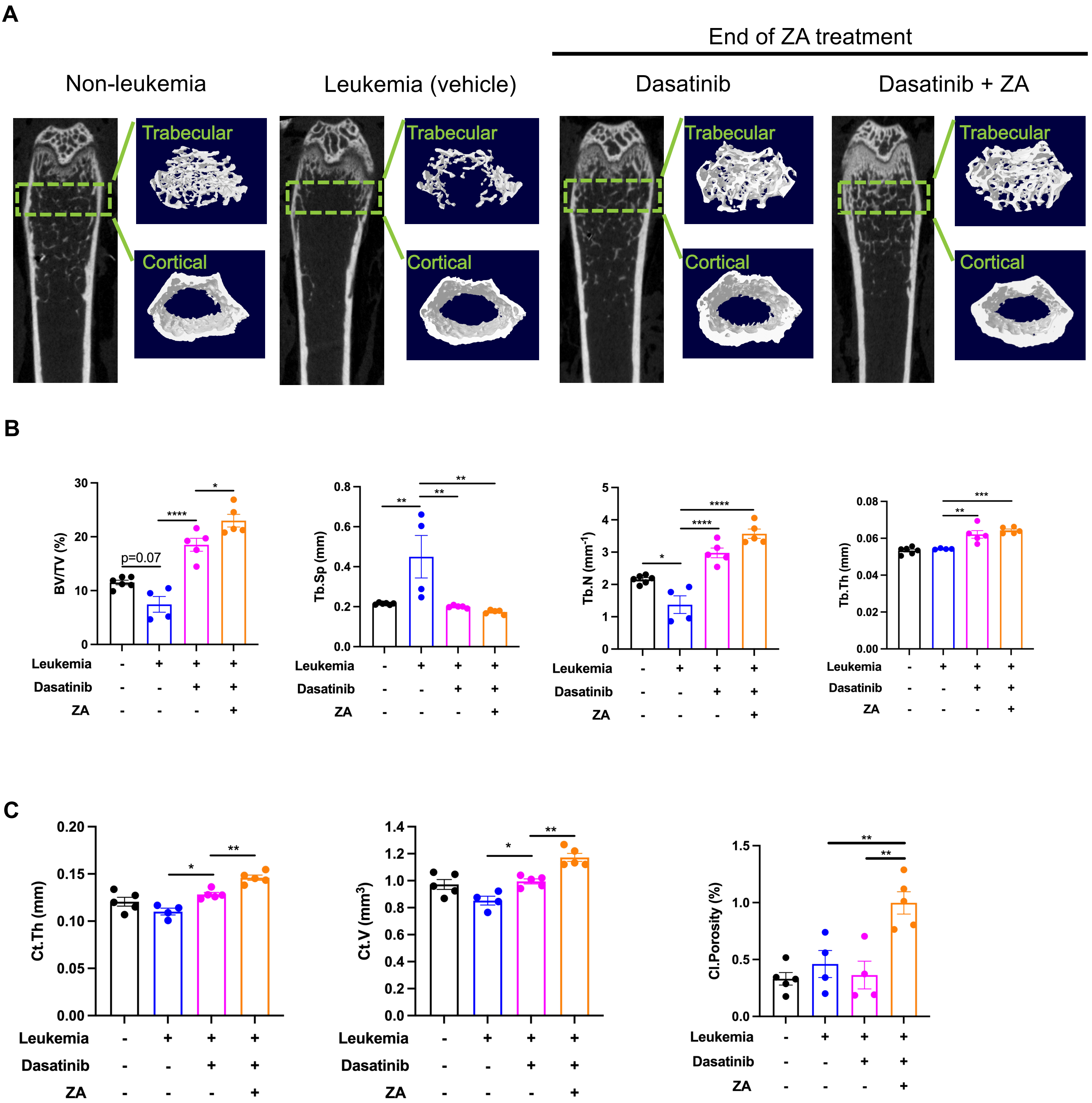

Supplementary Figure 1. Zoledronic acid (ZA) and dasatinib reduce bone loss in leukemia-bearing mice. Mice were treated with ZA for 2 weeks and dasatinib for 4 weeks commencing 13 days post-BCR-ABL1+ leukemia cell injection when the disease burden in the bone marrow was 34.2%±17.12%. Femurs were harvested when mice completed 2 weeks of treatment and micro-computed tomography (micro-CT) analysis was performed using a Skyscan 1176 micro-CT scanner. Age- and sex-matched non-leukemia mice were included in this analysis. (A) Micro-CT and 3D reconstruction images of the distal femur bone compartment. (B, C) Quantification of distal femur (B) trabecular bones and (C) cortical bones (n=4-6 mice per group). Error bars represented as mean ± SEM. *p<0.05, **p<0.01, ***p<0.001, ****p<0.0001 or the precise p-value where indicated. BV/TV, bone volume per trabecular volume; Tb.Sp, trabecular spacing; Tb.N, trabecular number; Tb.Th, trabecular thickness; Ct.Th, cortical thickness; Ct.V, cortical volume; Cl.Porosity, closed porosity.

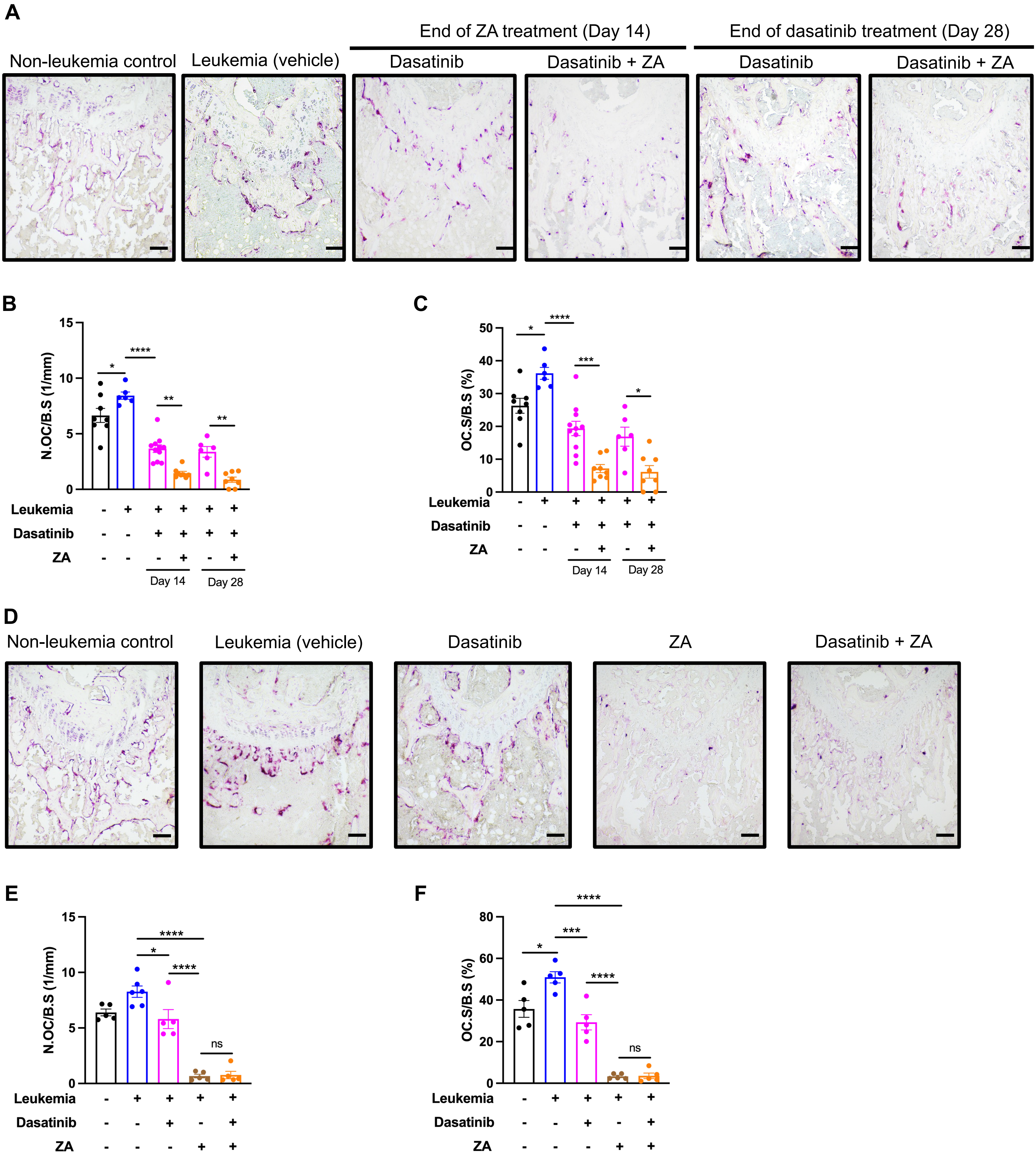

Supplementary Figure 2. Zoledronic acid (ZA) and dasatinib reduce the number of osteoclasts in leukemia-bearing mice. Mice were treated with ZA for 2 weeks and dasatinib for 4 weeks commencing 13 days post-BCR-ABL1+ leukemia cell injection when the disease burden in the bone marrow was 34.2%±17.12%. Age- and sex-matched non-leukemia mice were included in this analysis. Femurs were harvested (A-C) when mice completed 2 and 4 weeks of treatment and (D-F) when mice succumbed to disease after treatment. (A, D) Representative images of paraffin sections of the distal femur bone compartment stained for tartrate-resistant acid phosphatase (TRAP) (scale bar, 200µm). (B-C, E-F) Quantification of TRAP+ osteoclasts in the distal femur compartment (n=5-8 mice per group). One to two images per animal were analyzed. Error bars represented as mean ± SEM. *p<0.05, **p<0.01, ***p<0.001 and ****p<0.0001. N.OC/B.S, number of osteoclasts per bone surface; OC.S/B.S, osteoclast surface per bone surface.

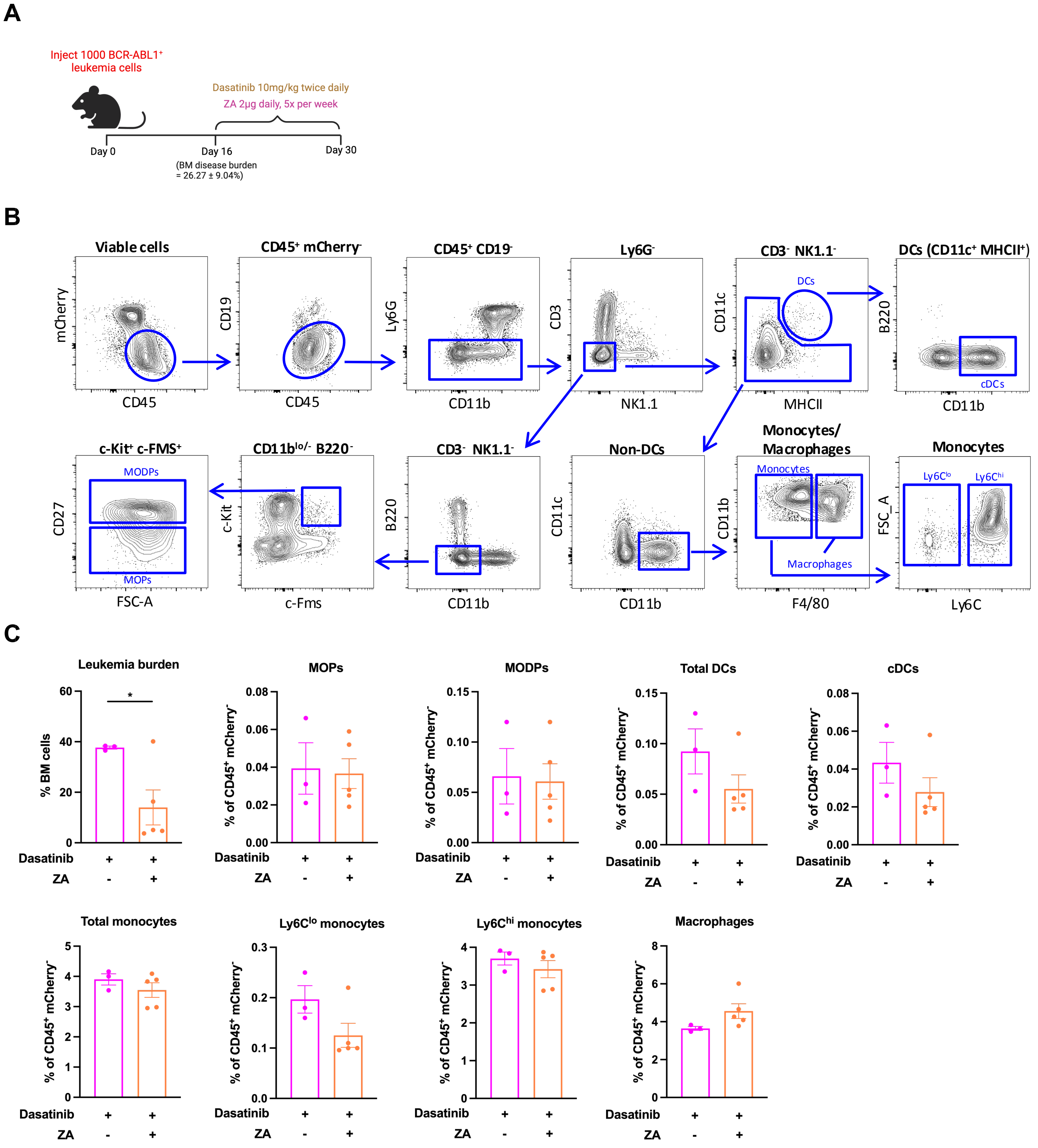

Supplementary Figure 3. Zoledronic acid (ZA) does not significantly change other monocyte-lineage populations in the bone marrow during dasatinib treatment. (A) Schematic diagram of the treatment schedule. Mice were treated with ZA and dasatinib for 2 weeks and femurs were harvested for analysis. (B) Flow cytometric gating strategy for populations of monocyte-lineage. (C) Percentage of monocyte-lineage populations after two weeks of treatment with dasatinib and ZA or dasatinib alone. MOPs (macrophage/osteoclast progenitors, $B220^-CD11b^{lo/-}c\text{-}Kit^+c\text{-}Fms^+CD27^{lo}$), MODPs (macrophage/osteoclast/dendritic cell progenitors, $B220^-CD11b^{lo/-}c\text{-}Kit^+c\text{-}Fms^+CD27^{hi}$), DCs (dendritic cells, $MHCII^+CD11c^+$), cDCs (conventional dendritic cells, $MHCII^+B220^-CD11c^+CD11b^+$), total monocytes ($CD11b^+F4/80^-$), $Ly6C^{lo}$ monocytes ($CD11b^+F4/80^-Ly6C^{lo}$), $Ly6C^{hi}$ monocytes ($CD11b^+F4/80^-Ly6C^{hi}$), macrophages ($CD11b^+F4/80^+$). n=3-5 mice per group. Error bars represented as mean ± SEM. *$p<0.05$.

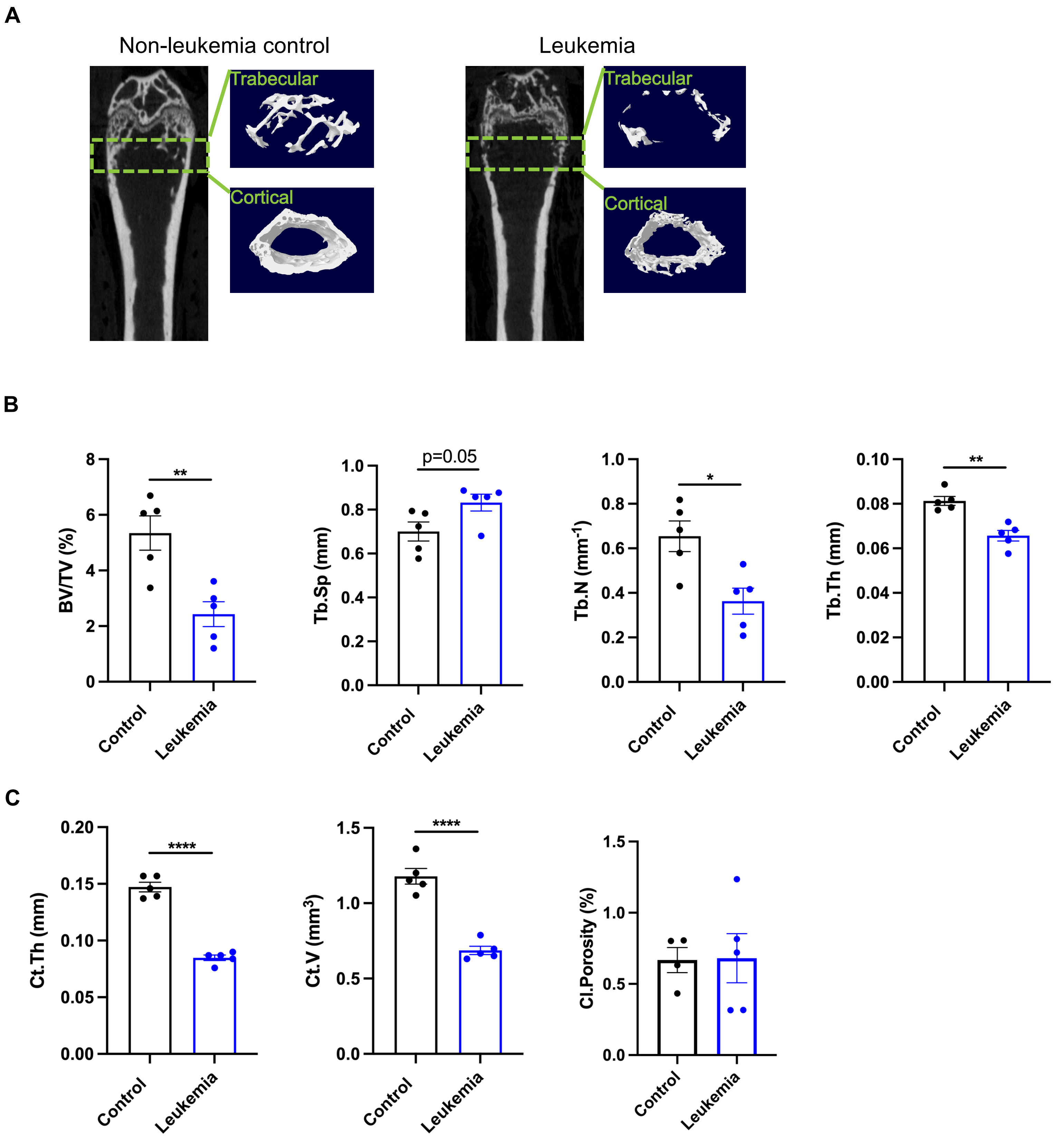

Supplementary Figure 4. Bone phenotype of the ALL-84 patient-derived xenograft model. Non-irradiated NSG mice were injected with ALL-84 cells. Femurs were harvested when mice succumbed to disease. (A) Micro-computed tomography and 3D reconstruction images of the distal femur bone compartment. (B, C) Quantification of distal femur (B) trabecular bones and (C) cortical bones (n=4-5 mice per group). Error bars represented as mean ± SEM. *p<0.05, **p<0.01, ****p<0.0001 or the precise p-value where indicated. BV/TV, bone volume per trabecular volume; Tb.Sp, trabecular spacing; Tb.N, trabecular number; Tb.Th, trabecular thickness; Ct.Th, cortical thickness; Ct.V, cortical volume; Cl.Porosity, closed porosity.

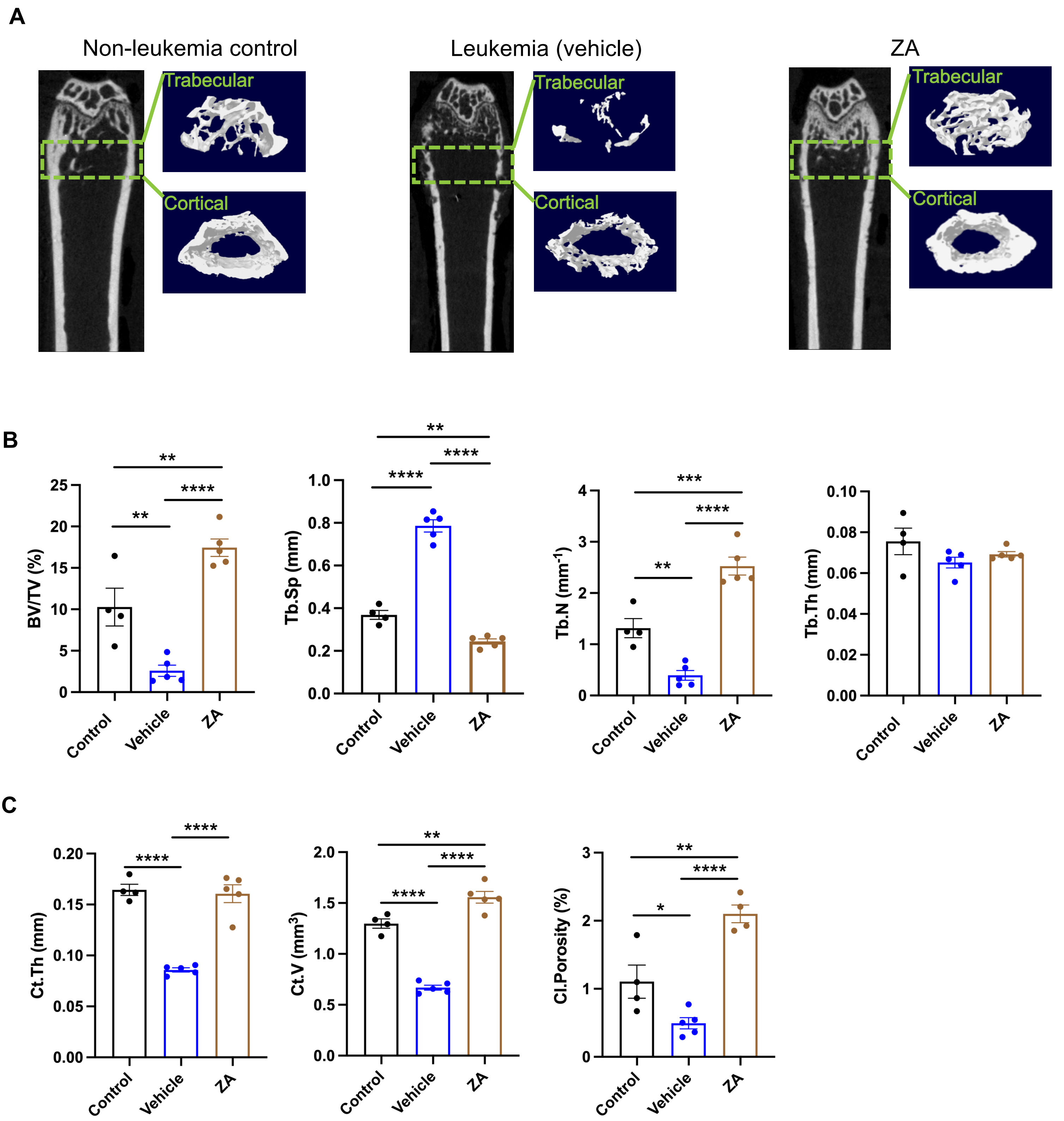

Supplementary Figure 5. Zoledronic acid (ZA) significantly reduces bone loss in the ALL-84 patient-derived xenograft model in the setting of low disease burden. Mice were treated with ZA for 2 weeks commencing 33 days post leukemia cell injection when the disease burden in the bone marrow was 7.13%±1.28%. Femurs were harvested when mice succumbed to disease after treatment and micro-computed tomography (micro-CT) analysis was performed using a Skyscan 1176 micro-CT scanner. Age- and sex-matched non-leukemia mice were included in this analysis. (A) Micro-CT and 3D reconstruction images of the distal femur bone compartment. (B,C) Quantification of distal femur (B) trabecular bones and (C) cortical bones (n=4-5 mice per group). Error bars represented as mean ± SEM. *p<0.05, **p<0.01, ***p<0.001 and ****p<0.0001. BV/TV, bone volume per trabecular volume; Tb.Sp, trabecular spacing; Tb.N, trabecular number; Tb.Th, trabecular thickness; Ct.Th, cortical thickness; Ct.V, cortical volume; Cl.Porosity, closed porosity.

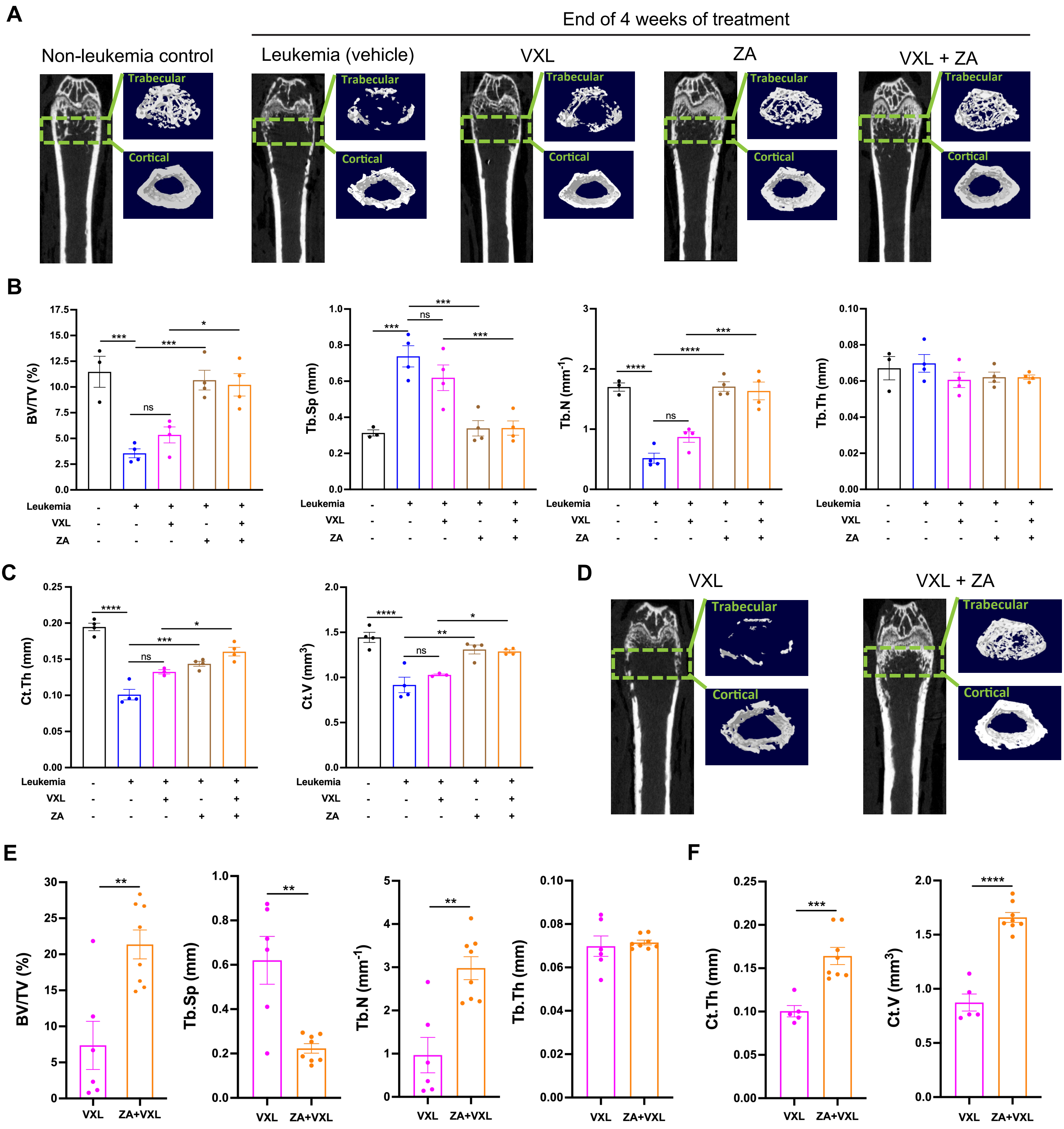

Supplementary Figure 6. Zoledronic acid (ZA) in combination with conventional chemotherapy significantly reduces bone loss in the ALL-84 patient-derived xenograft model in the setting of high disease burden. Mice were treated with ZA for 2 weeks and VXL (vincristine, dexamethasone and L-asparaginase) for 4 weeks commencing 55 days post leukemia cell injection when the disease burden in the bone marrow was 49.05%±17.47%. (A-C) Femurs were harvested when mice completed 4 weeks of treatment (n=3-4 mice per group) and (D-F) when mice succumbed to disease after treatment (n=6-8 mice per group). Micro-computed tomography (micro-CT) analysis was performed using a Skyscan 1176 micro-CT scanner. Age- and sex-matched non-leukemia mice were included in this analysis. (A, D) Micro-CT and 3D reconstruction images of the distal femur bone compartment. (B-C,E-F) Quantification of distal femur (D, G) trabecular bones and (E, H) cortical bones. Error bars represented as mean ± SEM. *p<0.05, **p<0.01, ***p<0.001 and ****p<0.0001. BV/TV, bone volume per trabecular volume; Tb.Sp, trabecular spacing; Tb.N, trabecular number; Tb.Th, trabecular thickness; Ct.Th, cortical thickness; Ct.V, cortical volume.

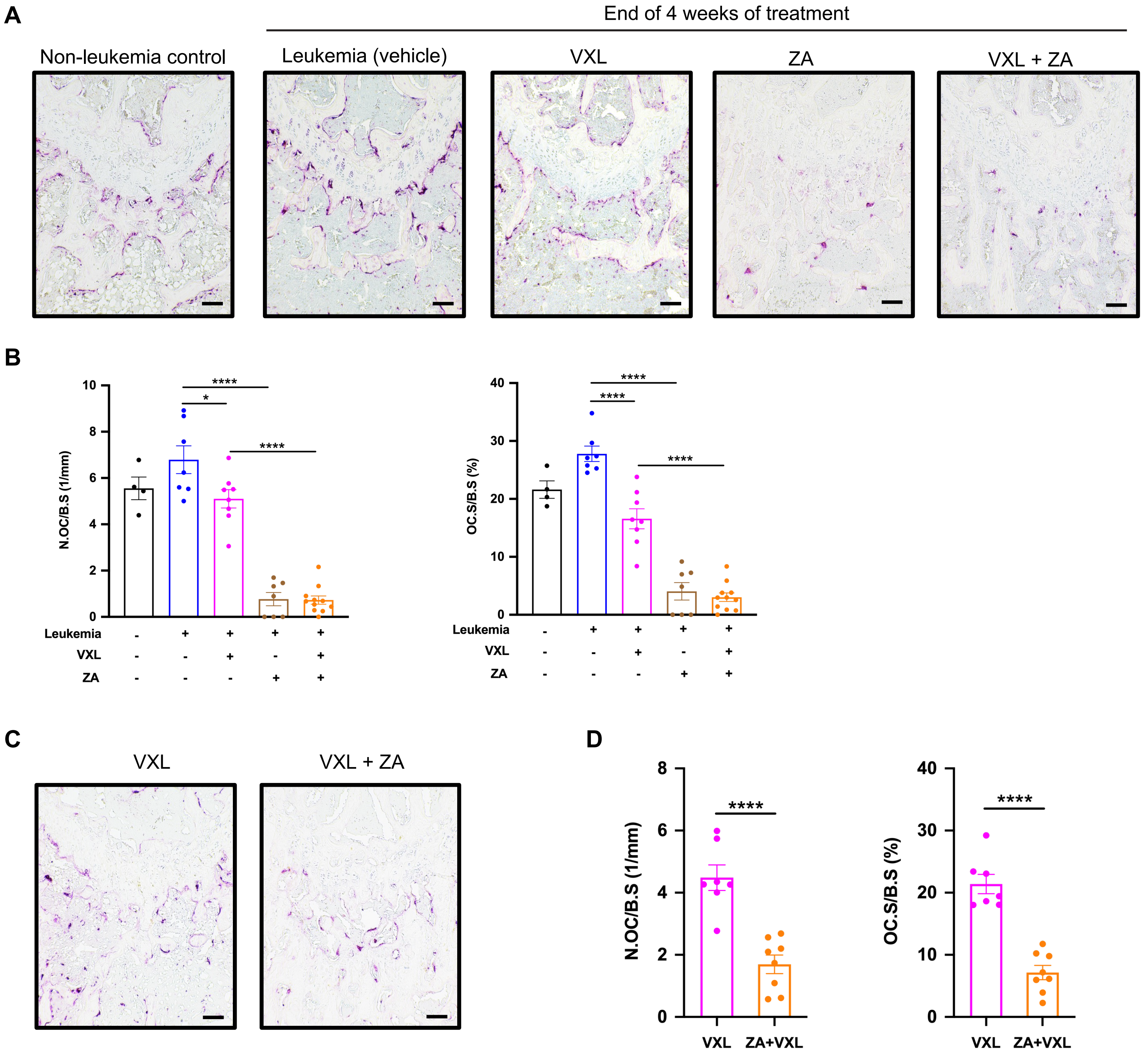

Supplementary Figure 7. Zoledronic acid (ZA) reduces the number of osteoclasts in the ALL-84 patient-derived xenograft model. Mice were treated with ZA for 2 weeks and VXL (viscristine, dexamethasone and L-asparaginase) for 4 weeks commencing 55 days post leukemia cell injection when the disease burden in the bone marrow was 49.05%±17.47%. Age- and sex-matched non-leukemia mice were included in this analysis. Femurs were harvested (A, B) when mice completed 4 weeks of treatment and (C, D) when mice succumbed to disease after treatment. (A, C) Representative images of paraffin sections of the distal femur bone compartment stained for tartrate-resistant acid phosphatase (TRAP) (scale bar, 200μm). (B, D) Quantification of TRAP+ osteoclasts in the distal femur compartment (n=4-8 mice per group). One to two images per animal were analyzed. Error bars represented as mean ± SEM. *p<0.05 and ****p<0.0001. N.OC/B.S, number of osteoclasts per bone surface; OC.S/B.S, osteoclast surface per bone surface.

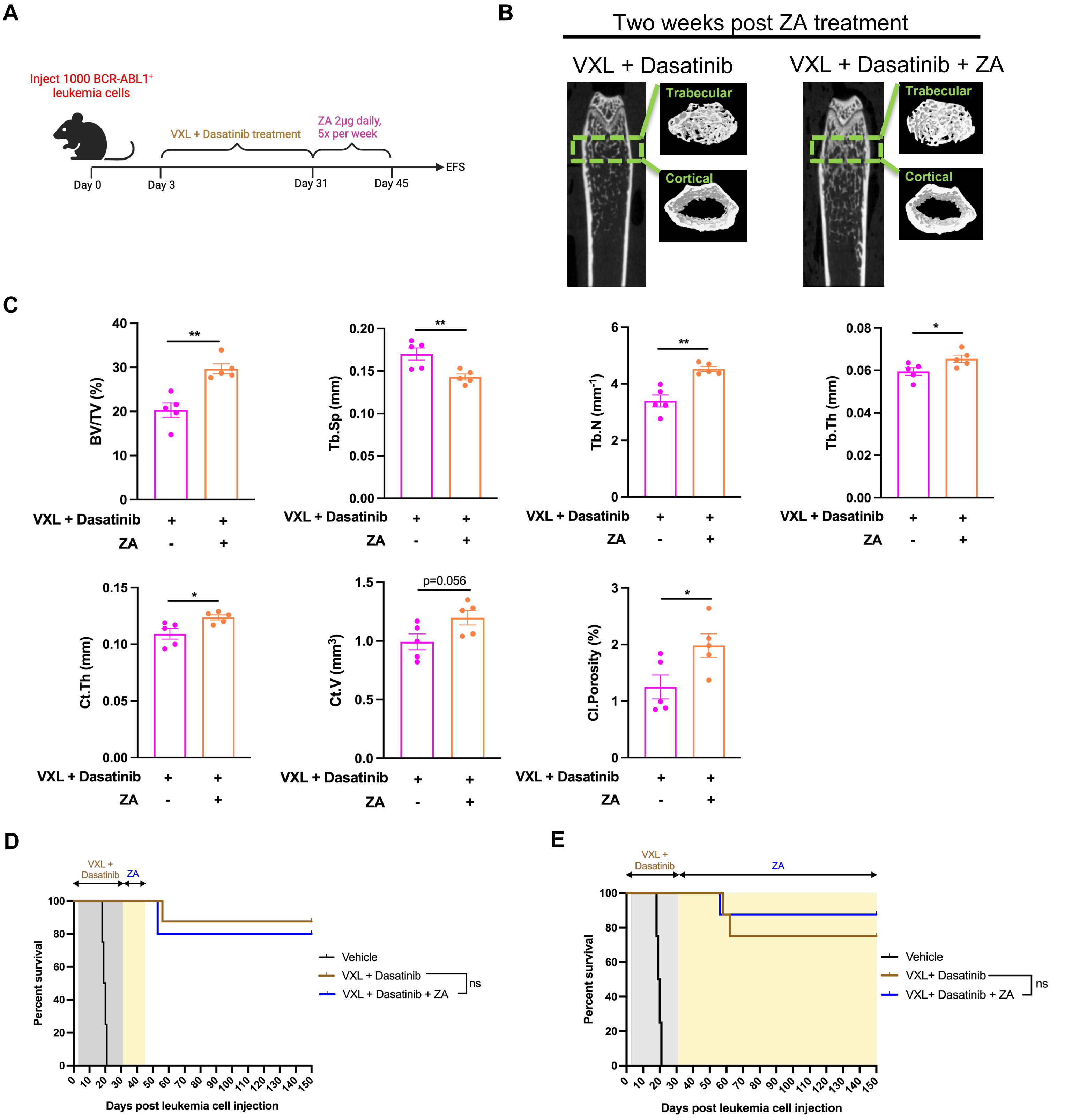

Supplementary Figure 8. Adminstation of zoledronic acid (ZA) immediately after induction therapy reverses bone loss and does not promote leukemia relapse. (A) Schematic diagram of the treatment schedule. Mice were treated with VXL (vincristine, dexamethasone and L-asparaginase) and dasatinib for 4 weeks followed by ZA for 2 weeks commencing 3 days post leukemia cell injection. (B) Micro-computed tomography and 3D reconstruction images of the distal femur bone compartment. (C) Quantification of distal femur trabecular bones and cortical bones (n=5 mice per group). Error bars represented as mean ± SEM. *p<0.05, **p<0.01, or the precise p-value where indicated. BV/TV, bone volume per trabecular volume; Tb.Sp, trabecular spacing; Tb.N, trabecular number; Ct.Th, cortical thickness; Ct.V, cortical volume; Cl.Porosity, closed porosity. (D, E) Kaplan-Meier curves representing event-free survival of leukemia-bearing mice receiving vehicle (n=4 mice per group) or VXL and dasatinib for 4 weeks commencing 3 days post leukemia cell injection followed by ZA administered for (D) two weeks or (E) as continuous treatment (n=8-10 mice per group). ns indicates not significant (p>0.05).

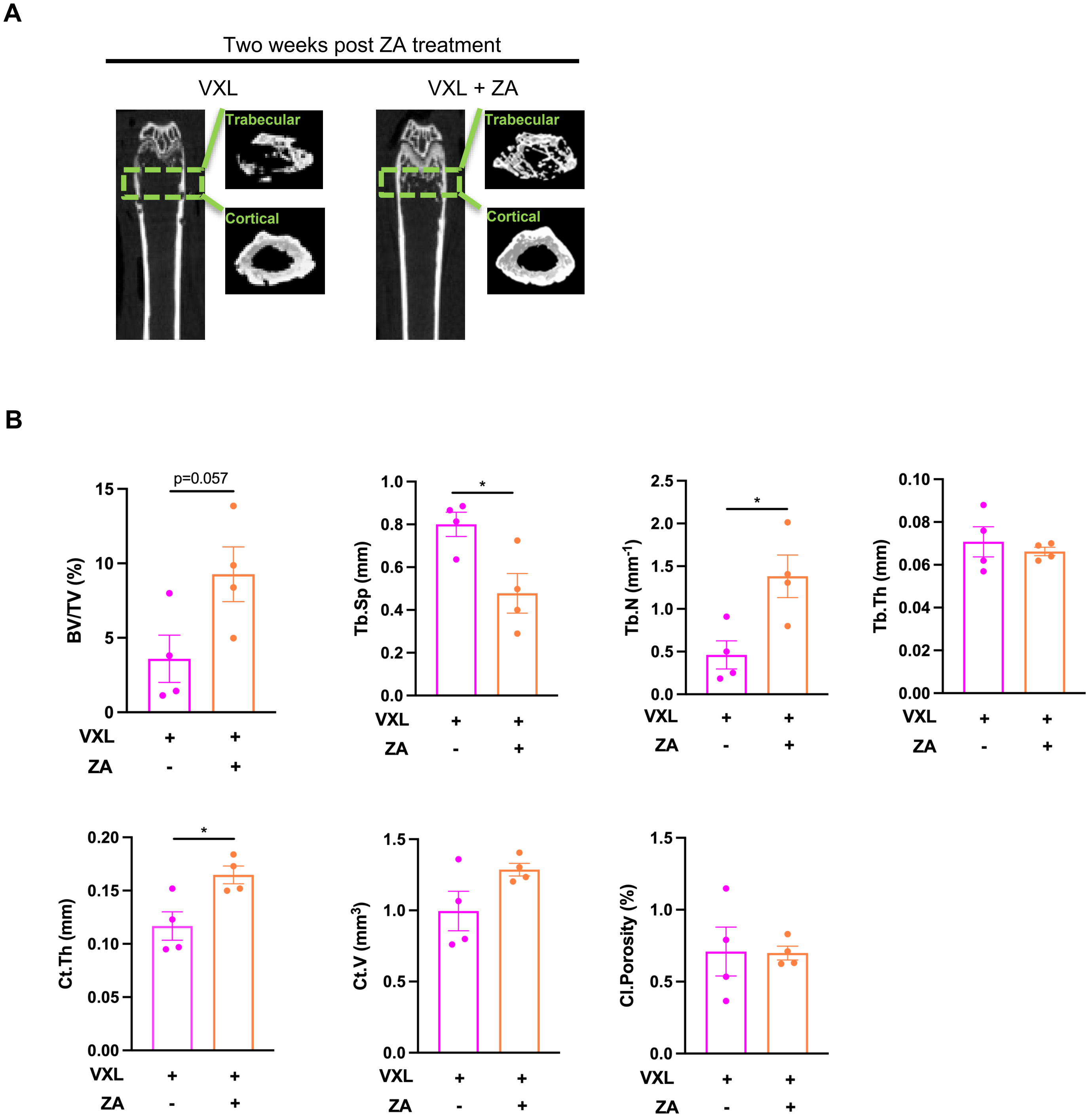

Supplementary Figure 9. Administration of zoledronic acid (ZA) after induction therapy significantly reduces bone loss in the ALL-84 patient-derived xenograft model. Mice were treated with VXL (vincristine, dexamethasone and L-asparaginase) for 4 weeks commencing 56 days post leukemia cell injection when the disease burden in the bone marrow was 41.83%±10.76% followed by ZA treatment for 2 weeks. Femurs were harvested 2 weeks post ZA treatment and micro-computed tomography (micro-CT) analysis was performed using a Skyscan 1176 micro-CT scanner. (A) Micro-CT and 3D reconstruction images of the distal femur bone compartment. (B) Quantification of distal femur trabecular bones and cortical bones (n=4 mice per group). Error bars represented as mean ± SEM. *$p<0.05$. BV/TV, bone volume per trabecular volume; Tb.Sp, trabecular spacing; Tb.N, trabecular number; Tb.Th, trabecular thickness; Ct.Th, cortical thickness; Ct.V, cortical volume; Cl.Porosity, closed porosity.

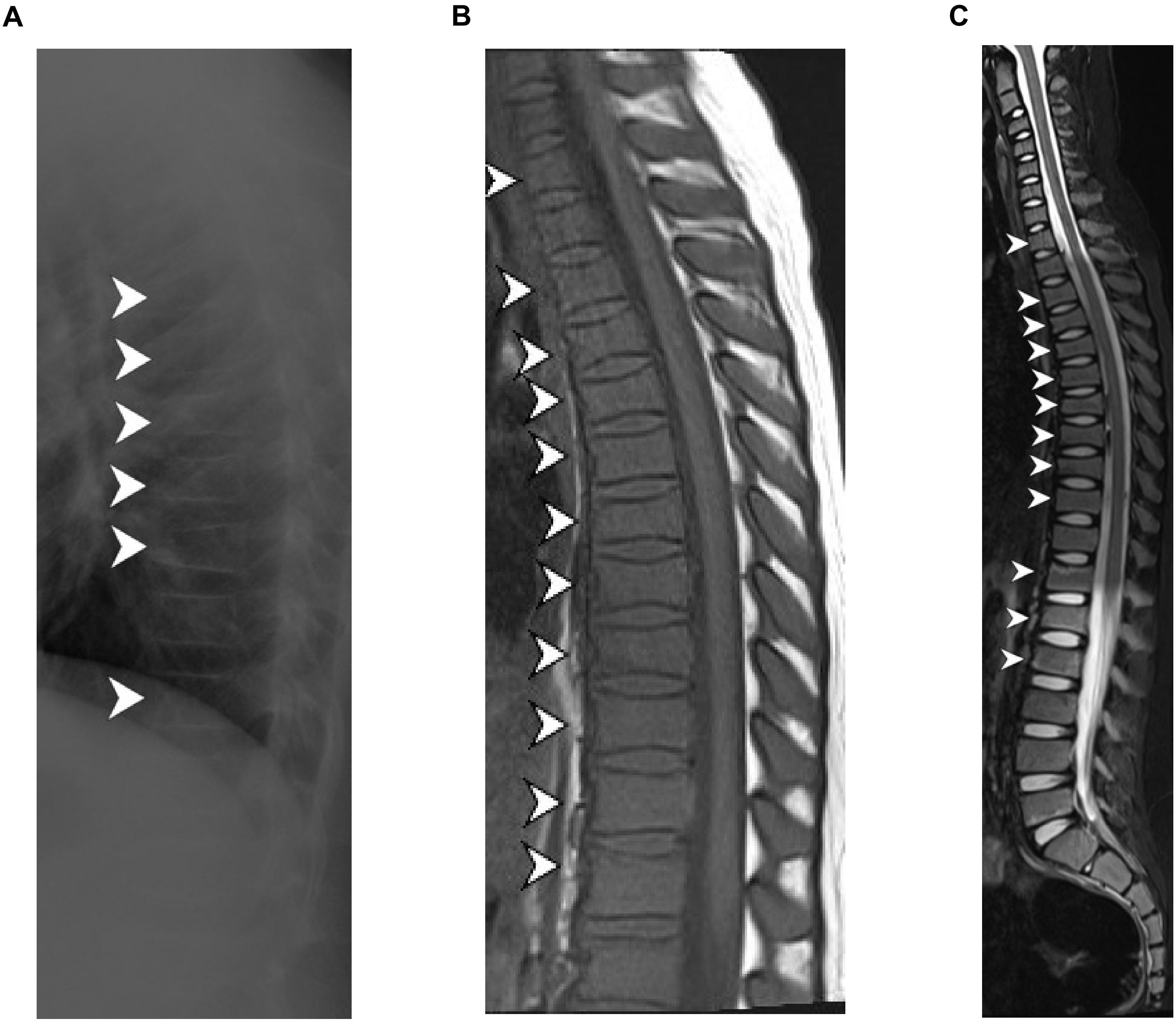

Supplementary Figure 10. Clinical case 1. Composite image of (A) lateral chest radiograph (B) sagittal T1 thoracic spine and (C) sagittal T2 TIRM whole spine MRI sequences showing diffuse marrow infiltration and multi-level vertebral body crush fractures (white arrowheads).

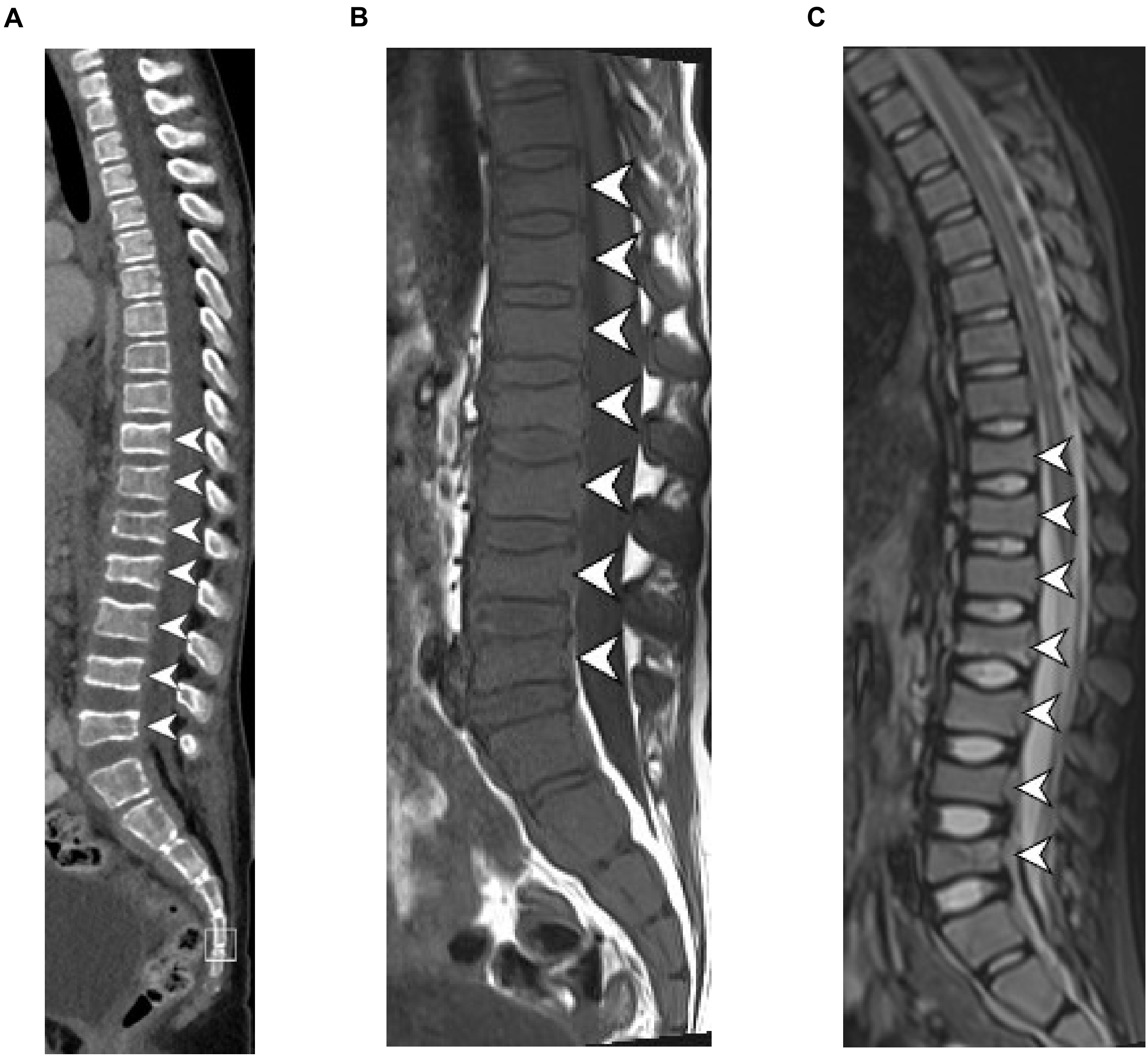

Supplementary Figure 11. Clinical case 2. Composite image of (A) sagittal CT reformats of the spine (B) sagittal T1 and (C) sagittal T2 TIRM MRI sequences of the lumbar spine showing diffuse marrow infiltration and multi-level vertebral body crush fractures (white arrowheads).

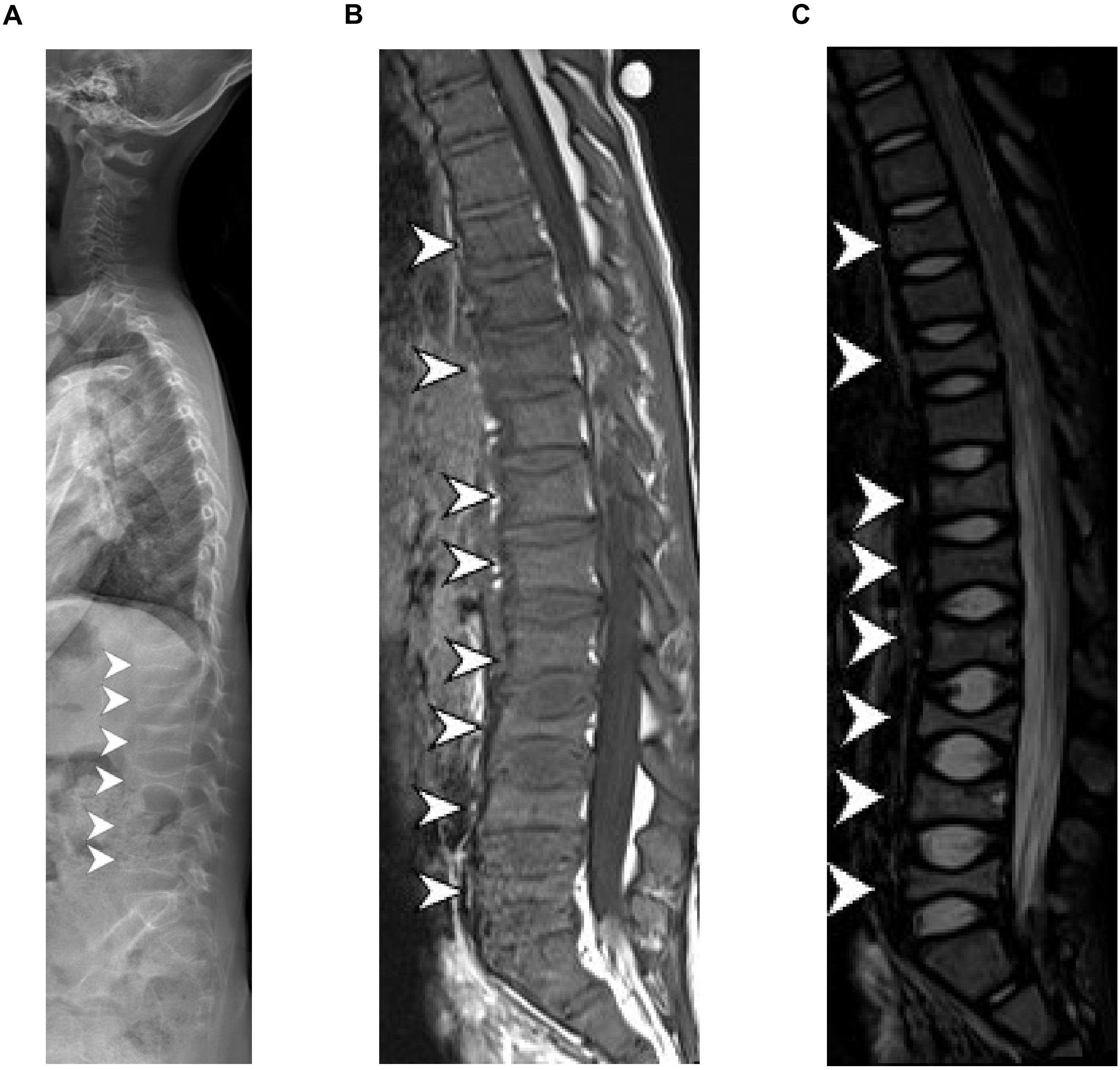

Supplementary Figure 12. Clinical case 3. Composite image of (A) lateral spine radiograph (B) sagittal T1 and (C) sagittal T2 STIR MRI sequences of the lumbar spine showing diffuse marrow infiltration and multi-level vertebral body crush fractures (white arrowheads).

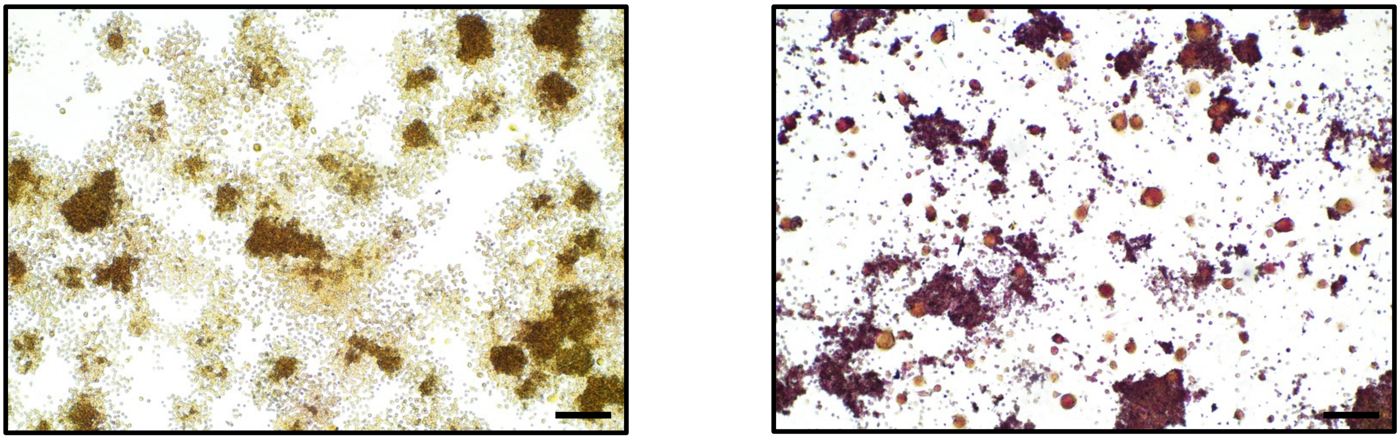

Supplementary Figure 13. Representative images showing differentiation of RAW264.7 cells into multinucleated osteoclasts following RANKL treatment. RAW264.7 cells were cultured with complete α-MEM in the absence (control, left) or presence (right) of human recombinant RANKL protein (50ng/ml) for 5 days (scale bar, 100μm). Formation of multinucleated osteoclasts following RANKL treatment was confirmed *via* tartrate-resistant acid phosphatase staining (scale bar, 100μm).

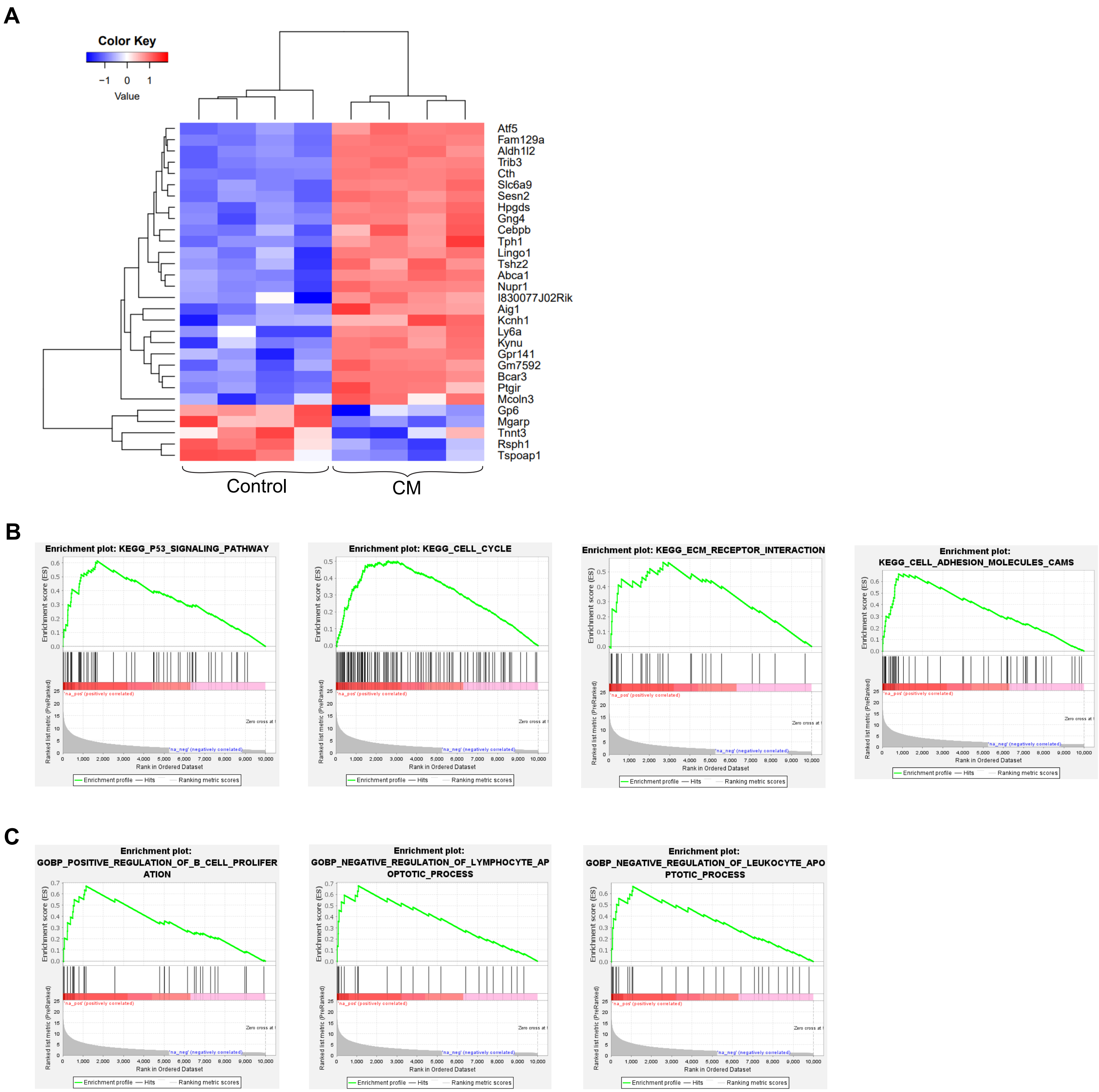

Supplementary Figure 14. Transcriptome analysis of leukemia cells cultured with conditioned media (CM) and control media. (A) Heatmap featuring normalized expression levels of the 30 differentially expressed genes identified at the 5% false discovery rate with a log fold-change value ≥1 or ≤-1 when comparing leukemia cells cultured in CM and control media. (B) Gene set enrichment plots from KEGG pathway analysis demonstrating significant upregulation in leukemia cells cultured in CM for the p53 signaling, cell cycle, extracellular matrix receptor interaction and cell adhesion molecules pathways. (C) Gene set enrichment plots from GO Biological Process analysis demonstrating significant upregulation in leukemia cells cultured in CM for positive regulation of B-cell proliferation and negative regulation of lymphocyte and leukocyte apoptotic process.
