# Supplementary material for: Targeting osteoclasts for treatment of high-risk B-cell acute lymphoblastic leukemia
Source: Blood Cancer J. 2025 Feb 27;15(1):25. doi: 10.1038/s41408-025-01239-3 (PMC11868389; doi:10.1038/s41408-025-01239-3)
Supplement: Supplementary file 2 — Supplementary Results [file 41408_2025_1239_MOESM2_ESM.pdf]

**Supplementary Results**

# Targeting osteoclasts for treatment of high-risk B-cell acute lymphoblastic leukemia

Rishi S Kotecha, Sarah M Trinder, Anastasia M Hughes, Benjamin H Mullin, Sarah Rashid, Jinbo Yuan, Jiake Xu, Owen Duncan, Patrycja Skut, Grace-Alyssa Chua, Sajla Singh, Joyce Oommen, Richard B Lock, Ursula R Kees, Sebastien Malinge, Vincent Kuek, and Laurence C Cheung

**Early administration of zoledronic acid in combination with chemotherapy was feasible and safe in three children with B-cell acute lymphoblastic leukemia**

The following three clinical cases suggest feasibility of safely administering zoledronic acid (ZA) in combination with chemotherapy during the early phases of therapy for B-cell acute lymphoblastic leukemia (B-ALL). This preliminary clinical data provides support for further investigation of our preclinical findings in future clinical trials.

**Clinical Case 1.** A 5-year-old boy presented to the emergency department in May 2022 with intermittent back and chest pain in the context of minor trauma. He was investigated with an X-ray and MRI of the spine, which demonstrated diffuse marrow signal abnormality and multi-level vertebral body crush fractures (Supplementary Figure 3). Bone densitometry scans demonstrated lumbar spine and left femoral neck Z-scores of -0.7 and -0.5 respectively. His initial full blood count demonstrated mild anemia with a hemoglobin of 104g/L (normal range 110-145g/L), a normal white blood cell count of 5.28 x $10^9$/L (normal range 5.00-17.00 x $10^9$/L), neutropenia of 0.53 x $10^9$/L (normal range 1.50-8.50 x $10^9$/L) and a normal platelet count of 190 x $10^9$/L (normal range 150-400 x $10^9$/L). A population of blasts accounting for 40% of nucleated cells was present in the peripheral film. A bone marrow aspirate confirmed a diagnosis of B-ALL on flow cytometry with a diagnostic lumbar puncture revealing CNS2a status. Cytogenetic analysis with G-band chromosomal analysis and fluorescent *in situ* hybridization (FISH) demonstrated a hyperdiploid karyotype with double trisomies of 4 and 10.

He was treated as per the standard risk Children's Oncology Group (COG) AALL0932 protocol and received a 0.025mg/kg intravenous infusion of ZA on day 1 of induction therapy. Oral calcium and vitamin D supplementation was administered for 14 days thereafter as part of standard institutional policy following ZA infusion. The infusion was given on the same day as placement of an implanted venous access device under general anesthetic. He developed a fever to 39.2°C (Grade 2 CTCAE), which was attributed to the general anesthetic and he was treated empirically with intravenous antibiotics until his blood cultures returned negative and he became afebrile. Three days following infusion, on day 3 of induction chemotherapy, he developed hypocalcemia (lowest ionized calcium 1.04mmol/L, Grade 2 CTCAE) that resolved with oral calcium supplementation and hypophosphatemia (lowest phosphate 0.38mmol/L, Grade 1 CTCAE) that resolved without intervention. There were no further adverse effects with potential attribution to ZA reported. A bone marrow aspirate at the end of induction demonstrated morphological remission and no evidence for minimal residual disease (MRD) (<0.01%) by flow cytometry.

From an orthopedic perspective, his spinal fractures were managed in a spinal brace to protect against further injury. Repeat MRI of the spine three months after commencing chemotherapy demonstrated stable appearances of the thoracic and lumbar spine with no further acute or interval compression fractures. His most recent bone densitometry scan performed in April 2024 revealed marked improvement in Z-scores of 2.8 and 2.0 for the spine and femur respectively. Ongoing serial surveillance X-rays of the spine have revealed no progressive features. He is currently receiving maintenance chemotherapy and is expected to finish treatment in July 2025.

**Clinical Case 2.** An 8-year-old boy presented with pancytopenia on a background history of extended oligoarticular juvenile idiopathic arthritis. He was initially diagnosed with juvenile idiopathic arthritis following presentation with isolated joint swelling post viral infection and was treated with intermittent oral steroid pulses and joint injections, eventually requiring oral methotrexate to induce remission. In December 2022, he developed progressive pain, poor mobility and pancytopenia ten months after initial presentation with a hemoglobin of 51g/L (normal range 115-155g/L), white blood cell count of 2.33 x $10^9$/L (normal range 5.00-17.00 x $10^9$/L) with an absolute neutrophil count of 0.92 x $10^9$/L (normal range 1.50-8.50 x $10^9$/L), and platelets of 130 x $10^9$/L (normal range 150-400 x $10^9$/L). Initial flow cytometry of the peripheral blood was not diagnostic of leukemia. However, MRI of the whole body and spine demonstrated diffuse marrow signal abnormality strongly suggestive of marrow infiltration and multi-level vertebral body compression fractures (Supplementary Figure 4). He proceeded to undergo a bone marrow aspirate which confirmed a diagnosis of B-ALL on flow cytometry, with a diagnostic lumbar puncture revealing CNS2a status. Cytogenetic analysis with G-band chromosomal analysis and FISH demonstrated a hyperdiploid karyotype.

The patient commenced treatment as per the high risk COG AALL1131 protocol. His bone marrow at the end of induction demonstrated morphological and MRD-negative (<0.01%) remission. Bone densitometry scans demonstrated globally reduced Z-scores of -1.4 for the whole body, -3.8 for the lumbar spine and -2.8 for the left femoral neck. He proceeded to receive 0.025mg/kg of intravenous ZA on day 33 of induction. This was tolerated without any adverse effects. He completed 14 days of oral calcium and vitamin D supplementation

following the ZA infusion and is currently receiving maintenance chemotherapy, with completion of therapy expected in April 2026.

**Clinical Case 3.** A 7-year-old boy who was following a strict dairy and gluten free diet, presented in September 2020 with two months of pain and poor mobility, with severe generalized osteopenia and multi-level vertebral body compression fractures identified on imaging (Supplementary Figure 5). Bone densitometry scans demonstrated severely reduced Z-scores, particularly of the lumbar spine (-5.1) and left femoral neck (-2.9). He had a background history of localized paratesticular embryonal rhabdomyosarcoma diagnosed at the age 4 years, treated as per Regimen A of the COG ARST0331 protocol.

He was initially managed by the orthopedic department with prolonged bed rest followed by spinal bracing. Five months after initial presentation, he received an initial dose of ZA at a dose of 0.05mg/kg. However, after receiving approximately half of the intended dose (~0.025mg/kg), he developed an acute phase reaction with low grade fever (CTCAE Grade 1), tachycardia (CTCAE Grade 1), tachypnoea (CTCAE Grade 1), vomiting (CTCAE Grade 1), hip pain (CTCAE Grade 1) and hypocalcemia (lowest ionized calcium 1.02mmol/L, CTCAE Grade 3), and the infusion was ceased. He received supportive care and calcium replacement and was hospitalized for three days. Following discharge, he was followed in the outpatient setting and remained on regular calcium and vitamin D supplementation.

At the time of bisphosphonate infusion, he was noted to have a mild normocytic anemia, which continued to progress over several months without apparent cause. He eventually developed pancytopenia eight months after initial presentation and underwent a bone

marrow aspirate in May 2021, which confirmed a diagnosis of B-ALL on flow cytometry with no evidence of central nervous system involvement (CNS1) following a diagnostic lumbar puncture. Cytogenetic analysis with G-band chromosomal analysis demonstrated a 47,XY,+5,del(6)(q13q22)[5]/46,XY[15] karyotype with no abnormalities identified on FISH.

Following diagnosis of B-ALL, he commenced treatment as per the high risk COG AALL1131 protocol, with prednisolone instead of dexamethasone in view of his profound osteopenia. His bone marrow at the end of induction demonstrated morphological remission but he remained MRD-positive at a level of 0.011% by flow cytometry. He was consequently escalated to the very high risk arm of the COG AALL1131 protocol. On day 2 of consolidation therapy, he received a further 0.05mg/kg of intravenous ZA in addition to oral calcium and vitamin D supplementation, which was tolerated without any adverse effects. He received a further dose of 0.05mg/kg of ZA on day 2 of the second cycle of maintenance therapy, which again was tolerated without incident. Repeat bone densitometry performed in June 2022 prior to this dose of ZA demonstrated a considerably improved lumbar spine Z-score of -2.3 and a stable left femoral neck Z-score of -3.2. Further bone densitometry performed in December 2023 showed ongoing improvement, with Z-scores of -0.5 and -1.2 for the spine and femur. There have been no progressive features on serial surveillance MRI scans and X-rays of the spine. He completed all planned treatment in September 2024 and remains in complete clinical and hematological remission.
