# Supplementary material for: Targeting osteoclasts for treatment of high-risk B-cell acute lymphoblastic leukemia
Source: Blood Cancer J. 2025 Feb 27;15(1):25. doi: 10.1038/s41408-025-01239-3 (PMC11868389; doi:10.1038/s41408-025-01239-3)
Supplement: Supplementary file 3 — Supplementary Methods [file 41408_2025_1239_MOESM3_ESM.pdf]

Supplementary Methods

Targeting osteoclasts for treatment of high-risk B-cell acute lymphoblastic leukemia

Rishi S Kotecha, Sarah M Trinder, Anastasia M Hughes, Benjamin H Mullin, Sarah Rashid, Jinbo Yuan, Jiake Xu, Owen Duncan, Patrycja Skut, Grace-Alyssa Chua, Sajla Singh, Joyce Oommen, Richard B Lock, Ursula R Kees, Sebastien Malinge, Vincent Kuek, and Laurence C Cheung

Ethics statement

Informed consent was obtained in accordance with the Declaration of Helsinki and the Human Research Ethics Committee of Perth Children's Hospital (RGS0000002372).

Animal models and drug treatment

Eight to 10-week old C57BL/6J and NOD-*scid* IL2Rgamma^{null} (NSG) mice were purchased from the Animal Research Centre, Perth. Animals were housed under pathogen-free conditions. The non-irradiated murine BCR-ABL1^{+} syngeneic B-cell acute lymphoblastic leukemia (B-ALL) model and the non-irradiated patient-derived xenograft model derived from a child with relapsed B-ALL (ALL-84) have been previously described.[1, 2] All control mice were age- and sex-matched. All animal studies were approved by the Animal Ethics Committee, The Kids Research Institute Australia, Perth (AEC#311). Mice were given a daily intraperitoneal injection of 2µg of zoledronic acid (ZA, Selleckchem, USA) in 100µL phosphate buffered saline (PBS) or 100µL of PBS alone, 5 days a week. ZA was administered for 2 weeks or as continuous treatment. For tyrosine kinase inhibitor treatment, mice were given 10mg/kg dasatinib (Selleckchem, USA), 100mg/kg imatinib (Selleckchem, USA) or vehicle *via* oral gavage twice daily for 28 consecutive days. For conventional chemotherapeutic treatment, mice received 0.15mg/kg vincristine (Pfizer, USA) once a week, 5mg/kg dexamethasone (Mylan, USA) once a day Monday to Friday, and 1000U/kg L-asparaginase (Kyowa Kirin, Japan) once a day Monday to Friday, for a total of 4 weeks *via* intraperitoneal injection. Control mice received vehicle treatment alone. Randomization was not used for the animal studies and the survival studies were not blinded.

Flow cytometry

Phenotypic analysis of mouse bone marrow (BM) monocyte-lineage populations was performed via flow cytometry on a BD FACSymphony™ A5 Cell Analyzer. Single cell suspensions of BM were prepared by flushing the femurs from BCR-ABL1^{+} mice with 2% fetal calf serum in PBS (2% FCS/PBS). Cells were then treated with Red Blood Cell Lysis Buffer (Thermo Fisher Scientific, USA) as previously described.[1] Cell numbers and viability were determined by staining the cells with ViaStain™ AOPI staining solution and analyzed via a Cellometer K2 Fluorescent Cell Counter (Nexcelom Bioscience, USA). BD Horizon™ Fixable Viability Stain 780 (BD Biosciences, USA) was used to exclude the dead cells. For phenotypic analysis of the monocytes, macrophages and dendritic cells (DCs), mononucleated cells were first stained with CD45-BV570 to separate the CD45^{+}mCherry^{-} hematopoietic cells. This was followed by staining with Ly6G-AF700, CD11b-BUV496, CD19-BV650, CD3-BUV737 and NK1.1-PE-Cy7 to exclude the granulocytes, B cells, T cells and NK cells. CD11c-BV711, MHCII-BV510 and B220-APC were used to identify the total DCs and conventional DCs.[1, 3] Ly6C-FITC and F4/80-BUV805 were used to distinguish monocytes (Ly6C^{lo} and Ly6C^{hi}) from the macrophages. Finally, c-Kit-BUV395, c-Fms-BV421 and CD27-BUV615 were used to identify the macrophage/osteoclast/DC progenitors (MODPs) and macrophage/osteoclast progenitors (MOPs).[3] Ly6C-FITC, CD45-BV570 and c-Fms-BV421 were purchased from BioLegend, USA. B220-APC was purchased from Thermo Fisher Scientific, USA. All other antibodies were purchased from BD Biosciences, USA.

Bone histomorphometry

To prepare paraffin-embedded bone sections, femurs were fixed in 4% paraformaldehyde (Sigma Aldrich, USA) in PBS at 4°C for 48 hours, followed by decalcification in 10% EDTA (Sigma

Aldrich, USA) at 4°C for 8 days. Bone samples were then processed through ethanol series for dehydration, xylene immersion and paraffin embedding. All tissue blocks were sectioned at a thickness of 5µm. For hematoxylin and eosin (H&E) staining, slides were incubated at 60°C for 45 minutes, deparaffinized using a Leica Autostainer XL (Leica micro-systems, Germany), and stained with H&E to visualize the gross anatomy of BM tissue. For staining of tartrate-resistant acid phosphatase (TRAP) enzymatic activity, deparaffinized slides were immersed in pre-warmed TRAP staining solution (50mM pH 5.2 sodium acetate, 0.15% naphthol AS-TR phosphate, 50mM sodium tartrate and 0.1% Fast Red TR, all reagents obtained from Sigma Aldrich, USA) at 37°C in the dark for 30-45 minutes, followed by counterstaining with hematoxylin (Vector Labs, USA). Images were captured using a Nikon Eclipse Ci Microscope fitted with a DS-L3 microscope control unit (Nikon, Japan). Osteoclast-specific parameters, including osteoclast surface per bone surface (OC.S/B.S) and number of osteoclasts per bone surface (N.OC/B.S), were quantified using Quantitative Pathology & Bioimage Analysis (QuPath) software as previously described.[4]

Micro-computed tomography (micro-CT) analysis

Murine, paraformaldehyde-fixed femurs were wrapped in PBS-moist tissues and immobilized in 2ml tubes (Eppendorf, Germany) for micro-CT scanning using a Skyscan 1176 micro-CT imaging system (Bruker, Belgium), as previously described.[1] In brief, bone samples were scanned using the following parameters: 50kV, 500µA, 1000ms, 0.5mm Al filter, 9µm pixel resolution, rotation step of 0.4°, frame averaging of 2. Volumetric images were reconstructed using NRecon software and orientated using DataViewer software. Analysis of bone parameters was performed using CTAn software. Three dimensional images of femurs were generated using CTVol software. All micro-CT analytic software were obtained from Bruker.

For metaphyseal trabecular bone analysis, a region beginning 0.5mm below the bottom of the growth plate and extending 1mm proximally, excluding the cortical bone, was selected as the volume of interest. For metaphyseal cortical bone analysis, the same region excluding trabecular bone was selected as the volume of interest. All micro-CT procedures and data analyses were performed at the Centre for Microscopy, Characterisation and Analysis (CMCA), University of Western Australia.

In vitro studies

RAW264.7 cells were cultured and maintained in α-MEM (Thermo Fisher Scientific, USA) supplemented with 10% fetal calf serum (CellSera, Australia), 1% Penicillin/Streptomycin (Thermo Fisher Scientific, USA) and 2mM L-glutamine (Sigma-Aldrich, USA) (*i.e.*, complete α-MEM) at 37°C, 5% CO_2 in accordance with a previously published protocol.[5] In brief, cells were seeded into 6-well cell culture plates (Nunclon, Thermo Fisher Scientific, USA) at a density of 5 x 10^4 cells per well. The next day, cells were treated with human recombinant receptor activator of nuclear factor kappa-B ligand (RANKL) protein (Thermo Fisher Scientific, USA) at a concentration of 50ng/ml for 5 days to induce osteoclastogenesis. Cell culture medium was replaced with fresh medium in the presence of human RANKL protein every 1-2 days. On day 5 post-differentiation, cells were fixed with 2.5% glutaraldehyde (Sigma Aldrich, USA), followed by TRAP staining to confirm formation of multinucleated osteoclasts as previously described.[1]

Proteomic analysis

Osteoclast-derived conditioned media (CM) was collected from RAW264.7 cells treated with 50ng/ml human RANKL protein for 5 days. Complete α-MEM exposed to the same incubation

conditions (*i.e.,* 37°C, 5% CO_2) but without cultured cells was used as control media. All CM were cleared of debris by centrifugation, followed by albumin-depletion using the ProMax Albumin Removal Kit (Polysciences, USA), as previously described.[6] In brief, 500µl of CM was added to 1750µl ProMax Binding/Wash Buffer and 100µl Promax particles. Samples were incubated for 20 minutes at room temperature, with gentle mixing. Particles were collected via magnetic separation, followed by three washes with 2.5ml ProMax Binding/Wash Buffer. Washed particles were then mixed in 500µl of 100mM, pH 8.5 tris (hydroxymethyl) aminomethane hydrochloric acid (VWR Life Sciences, USA) and 2% sodium dodecyl sulfate (Sigma Aldrich, USA) for 10 minutes at room temperature to elute proteins. Particles were then magnetically separated from supernatants containing eluted proteins, with cleared supernatants frozen at -80°C until further proteomic analysis.

Protein was purified and digested by SP3[7] using carboxylated beads (Cytiva, USA) at a ratio of 1.8µg beads/µl. Samples were analyzed over 90 minutes in 0-35% acetonitrile (0.1% formic acid) using a U3000 RSLC nano liquid chromatograph coupled to an Orbitrap Exploris 480 (Thermo Fisher Scientific, USA). Chromatography was conducted by direct injection on approximately 200mm of 1.9µm Reprosil Pur C18 (Dr Maisch, Germany) packed in a 75µm fritted, pulled emitter column at a flow rate of 0.25µl/minute. Full scan mass spectrometry (MS) was conducted at 120k resolution with intensity-based data dependent MS/MS sampling performed for 1.5 seconds following each MS scan. Data files were analyzed by Maxquant v2.1.4.0 with match-between-runs enabled. A combination of Uniprot UP000005640 (Human) and UP000009136 (Bovine) protein sequences were used for matching experimental spectra.

Cell cycle and apoptosis assays

PER-M60 cells[1] were seeded in 24-well cell culture plates (Nunclon, Thermo Fisher Scientific, USA) at a density of 3 x 10^4 cells per 250µl of complete α-MEM. The cells were then resuspended in either 250µl of osteoclast-derived CM or control media and cultured for 3 days at 37°C, 5% CO_2. To quantify cell proliferation, we first stained the cells with ViaStain™ AOPI Staining Solution (Nexcelom Bioscience, USA), followed by measurement of cell count using a Cellometer (Nexcelom Bioscience, USA). For cell cycle evaluation, PER-M60 cells cultured in osteoclast-derived CM or control media were fixed and washed using BD Cytofix/Cytoperm™ solution (BD Biosciences, USA) according to manufacturer's instructions. Cells were then stained with Hoechst 33342 (BD Biosciences, USA) at 20µg/ml and anti-Ki-67-BV786 (BD Biosciences, USA) at 1:20 for 30 minutes in the dark. Flow cytometry was performed using a BD LSRFortessa™ X-20 for cell cycle analysis. Different cell cycle phases (G0, G1, S/G2/M) were gated as previously described.[8] For evaluation of apoptosis, cells were processed using a FITC Annexin V Apoptosis Detection Kit I (BD Biosciences, USA) and co-stained with DAPI at 0.2µg/ml (Thermo Fisher Scientific, USA). Apoptotic cells were quantified using a BD LSRFortessa™ X-20.

RNA sequencing

PER-M60 cells were seeded at a density of 3 x 10^4 cells per well in a 24-well cell culture plate (Nunclon, Thermo Fisher Scientific, USA) in complete α-MEM. Osteoclast-derived CM collected from differentiated RAW264.7 cells was added to the PER-M60 cells at a ratio of 1:1 complete α-MEM:osteoclast-derived CM. Complete α-MEM was used as control. After 3 days, the cells were harvested and RNA was extracted using the RNeasy Plus Mini Kit (Qiagen, Germany) according to manufacturer's instructions. Stranded PolyA RNAseq libraries were

prepared using the Sureselect HS2 library preparation kit (Agilent Technologies, USA). Total RNA quantity and purity was analyzed using a Tapestation 4200 and Qubit, with all samples demonstrating an RNA integrity number of ≥9. Samples were sequenced on a NovaSeq 6000 (Illumina, USA) at 2 X 50 cycles, with approximately 20 million raw paired-end reads generated per sample. Raw sequencing data is available *via* the Gene Expression Omnibus (GEO) database under the accession number GSE220262.

Reads were aligned to the mouse genome using STAR v2.7.5a and raw read counts were summarized at the gene level (mm10 GENCODE Genes M25). Genes with very low expression (<1 count per million in 4 or more samples) were removed from the dataset. Normalization of the RNAseq data was performed using the trimmed mean of M-values method, with subsequent differential gene expression analysis completed using the quasi-likelihood F-test function in edgeR.[9] Correction for multiple testing was performed using the Benjamini-Hochberg procedure. Plots were generated in R using the 'ggplot2' (volcano and bubble plots) and 'gplots' (heatmap) packages.

Statistical analysis

Statistical analyses and graphics were performed using GraphPad Prism version 9.4.1 (GraphPad Software, USA). For comparison between two groups, data were analyzed using the two-tailed unpaired Student's t-test. Groups were compared using one-way ANOVA with Tukey's HSD post-hoc test for comparison of 3 groups or more. Survival studies were analyzed using log-rank test. The interquartile range method was used to determine any outliers. Statistical tests were not used to determine sample size. The results are presented as mean ± standard error of mean (SEM). A p-value <0.05 was considered statistically significant.

References

1. Cheung LC, Tickner J, Hughes AM, et al. New therapeutic opportunities from dissecting the pre-B leukemia bone marrow microenvironment. *Leukemia*. May 8 2018;32(11):2326-2338. doi:10.1038/s41375-018-0144-7

2. Rokita JL, Rathi KS, Cardenas MF, et al. Genomic profiling of childhood tumor patient-derived xenograft models to enable rational clinical trial design. *Cell Rep*. Nov 5 2019;29(6):1675-1689.e9. doi:10.1016/j.celrep.2019.09.071

3. Xiao Y, Palomero J, Grabowska J, et al. Macrophages and osteoclasts stem from a bipotent progenitor downstream of a macrophage/osteoclast/dendritic cell progenitor. *Blood Adv*. Oct 24 2017;1(23):1993-2006. doi:10.1182/bloodadvances.2017008540

4. Chen K, Liao S, Li Y, et al. Osteoblast-derived EGFL6 couples angiogenesis to osteogenesis during bone repair. *Theranostics*. 2021;11(20):9738-9751. doi:10.7150/thno.60902

5. Huang XL, Huang LY, Cheng YT, et al. Zoledronic acid inhibits osteoclast differentiation and function through the regulation of NF-kappaB and JNK signalling pathways. *Int J Mol Med*. Aug 2019;44(2):582-592. doi:10.3892/ijmm.2019.4207

6. Nakamura R, Nakajima D, Sato H, Endo Y, Ohara O, Kawashima Y. A simple method for in-depth proteome analysis of mammalian cell culture conditioned media containing fetal bovine serum. *Int J Mol Med*. Mar 4 2021;22(5)doi:10.3390/ijms22052565

7. Hughes CS, Moggridge S, Muller T, Sorensen PH, Morin GB, Krijgsveld J. Single-pot, solid-phase-enhanced sample preparation for proteomics experiments. *Nat Protoc*. Jan 2019;14(1):68-85. doi:10.1038/s41596-018-0082-x

8. Wilson A, Murphy MJ, Oskarsson T, et al. c-Myc controls the balance between hematopoietic stem cell self-renewal and differentiation. *Genes Dev*. Nov 15 2004;18(22):2747-63. doi:10.1101/gad.313104

9. Robinson MD, McCarthy DJ, Smyth GK. edgeR: a Bioconductor package for differential expression analysis of digital gene expression data. *Bioinformatics*. Jan 1 2010;26(1):139-40. doi:10.1093/bioinformatics/btp616
